# Locked and (Un)-Loaded Discussions: A Pediatric Resident Safe Firearm Storage Counseling Curriculum

**DOI:** 10.15766/mep_2374-8265.11028

**Published:** 2020-12-04

**Authors:** Cody Clary, Lindsey Lambarth, Ruchi Kaushik

**Affiliations:** 1 Resident, Department of Pediatrics, Baylor College of Medicine and Children's Hospital of San Antonio; 2 Assistant Professor, Department of Pediatrics, Baylor College of Medicine and Children's Hospital of San Antonio

**Keywords:** Firearms, Safe Storage, Counseling, Curriculum, Simulation, Advocacy

## Abstract

**Introduction:**

Firearm-related fatalities are a public health crisis. Despite recognizing the vital nature of counseling parents/caregivers regarding firearm safety, residents remain uncomfortable asking patients about the presence of firearms in homes and discussing American Academy of Pediatrics recommendations regarding safe firearm storage.

**Methods:**

We designed an interactive curriculum to improve pediatric resident knowledge, skills, attitudes, and behavior regarding counseling families about safe firearm storage. Components of the curriculum included a didactic session, a hands-on experience to better understand the parts of a firearm and its relevant storage/safety devices, and role-playing scenarios.

**Results:**

The curriculum was delivered to 53 pediatric residents in two different residency programs. A statistically significant improvement in knowledge and skills related to safe firearm storage counseling was demonstrated in both settings. Furthermore, a statistically significant change in counseling behavior was noted among one resident group. Curriculum evaluation revealed overwhelmingly positive learner responses.

**Discussion:**

An adaptable interactive safe firearm storage counseling curriculum was well received by pediatric residents and improved resident knowledge and skills, resulting in an increase in safe firearm storage counseling discussions with families.

## Educational Objectives

After completion of this workshop, learners will be able to:

1. Describe the current status of firearm injuries in the US.
2. State the American Academy of Pediatrics policy on firearms and explain the safest methods to secure firearms in the home and vehicles.
3. Identify the various parts of a firearm and related safety and storage devices.
4. Apply this new knowledge in a clinical setting to educate families about safe storage of firearms.

## Introduction

Firearm-related fatalities, including homicide, suicide, and unintentional weapon discharges, are one of the top three causes of death among children and adolescents in the United States.^1^ More than one-fourth of all deaths are firearm related for individuals 15–19 years of age. Moreover, firearm injuries are the leading cause of death among Black males 15–34 years of age.

Suicide is the second most common cause of death among adolescents 15–19 years of age.^2^ Suicide rates among American children and adolescents are eight times those of other high-income countries, and the ease of access to firearms in the US considerably increases the risk of youth suicide.^3^ Risk of fatal suicide is statistically significantly higher in homes with firearms as compared to homes that do not have a firearm.^4^ While having a firearm does not increase the risk of suicidal ideation, a child with suicidal ideation is seven times more likely to have a plan involving firearms if there is one in the home, and children and adolescents often choose this method because of its high success rate.^5^ Typically, suicide among children and adolescents is an impulsive action due to personal life stressors, but the lethality of firearms prevents a second chance for those who may have considered changing their minds.^4^

Firearm-related deaths also include incidents resulting from unintentional firearm discharges.^1^ Children 5–14 years of age in the US are 10 times as likely to die of unintentional injuries as compared to children in other high-income countries. In 2019, 1,837 unintentional firearm discharges occurred in the US, a 10% increase over this statistic in 2018.^6^ These unacceptably high rates are likely due to easy child access to firearms, lack of proper gun storage, and absence of safety mechanisms on several firearms.

The American Academy of Pediatrics (AAP) asserts that the safest home is a home without a firearm.^1^ As compared to homes without firearms, families in homes with a firearm have a higher probability of experiencing a homicide, suicide, or unintentional firearm injury that results in death.^7^ If a family chooses to own a firearm, the AAP recommends it be stored locked in a safe and unloaded, with the ammunition locked in a separate location.^1^

Despite these storage recommendations, physicians do not believe that patients would accept their anticipatory guidance on this topic,^8^ and many pediatric residents and practicing physicians are not comfortable asking patients about the presence of firearms in homes^9,10^ and are not providing this counseling.^9–11^ Interestingly, however, the majority of patients believe it is acceptable for physicians to discuss safe firearm storage, and a review of the literature suggests physicians’ beliefs that counseling would not result in behavior change are unfounded.^12^

Baylor College of Medicine-Children's Hospital of San Antonio (BCM-CHofSA) pediatric residents received no formal education in safe firearm storage counseling prior to implementation of this curriculum. The need for such a formal curriculum was revealed by the fact that between May 1 and June 15, 2019, only roughly 8% of well-child care visits for patients less than 3 years of age completed by pediatric residents included firearm safety discussions.

The nonpartisan Be SMART program is a national, nonprofit organization dedicated to eliminating unintentional firearm injuries through safe firearm storage conversations.^13^ The SMART acronym represents securing guns in homes and vehicles, modeling responsible behavior around guns, asking about unsecured guns in other homes, recognizing the risks of child and teen suicide, and telling peers to Be SMART.

To address the lack of comfort and the misconceptions described in the literature, the BCM-CHofSA residency program collaborated with the San Antonio Be SMART chapter and local law enforcement to develop a curriculum to improve the safe firearm storage counseling knowledge, skills, attitudes, and behaviors of pediatric residents. To our knowledge, such a curriculum is not currently available in *MedEdPORTAL* or *MedEdPublish*.

## Methods

### Setting

Pediatric residents were the target learners of this intervention. We delivered the curriculum to all BCM-CHofSA pediatric residents consenting to participate during a 180-minute didactic session in the first iteration. For the second iteration, the Brooke Army Medical Center (BAMC) pediatric residency program invited us to deliver the curriculum during a 90-minute teaching session.

### Curriculum Design

The design and elements of the curriculum are depicted in Table 1. Curricular components included the following:

• Pediatric residents completed a brief, preintervention survey (Appendix A) to assess baseline knowledge, skills, and attitudes about safe firearm storage counseling with parents/caregivers (5 minutes).
• We, the principal investigators, presented a didactic lecture to describe the current status of firearm injuries in the US, stated the AAP policy statement on firearms, and explained the safest methods to secure firearms in the home and vehicles (Appendix B). This portion of the curriculum included a brief description of the use of conflict resolution strategies when conducting safe firearm storage counseling discussions with parents/caregivers (30 minutes).^14^
• A session devoted to identifying the various parts of a firearm and related safety and storage devices followed the didactic lecture. We invited a member of a local medical school campus’ law enforcement to present this portion of the curriculum. Educators are encouraged to develop a relationship with their own local law enforcement entities to implement this critical piece of the curriculum. However, in the event that this is not feasible, a video file has been included (Appendix C). We drafted a sample phone or email script for programs to engage their own local law enforcement in the delivery of this portion of the session (Appendix D). Because of time constraints, BAMC pediatric residents did not participate in this element of the curriculum (live: 30 minutes; video: 15 minutes).
• We then conducted a role-playing session aimed at improving pediatric resident skills and applying new knowledge in a clinical setting to educate families about safe storage of firearms utilizing three scenarios (Appendix E; see also the facilitator guide for implementing the role-playing scenarios in Appendix F). We asked residents to assemble into small groups and practice safe firearm storage counseling using realistic clinic scenarios (45 minutes to conduct three scenarios followed by a debriefing session).
• Following delivery of the curriculum, pediatric residents completed a brief postintervention survey (Appendix G) to reassess knowledge, skills, and attitudes about safe firearm storage counseling with parents/caregivers. Additionally, we incorporated questions to evaluate the curriculum design and to elicit resident comments regarding strengths and opportunities in an effort to reinforce the continued implementation of curricular components that were effective and make adjustments to those that were not (5 minutes).

**Table 1.**
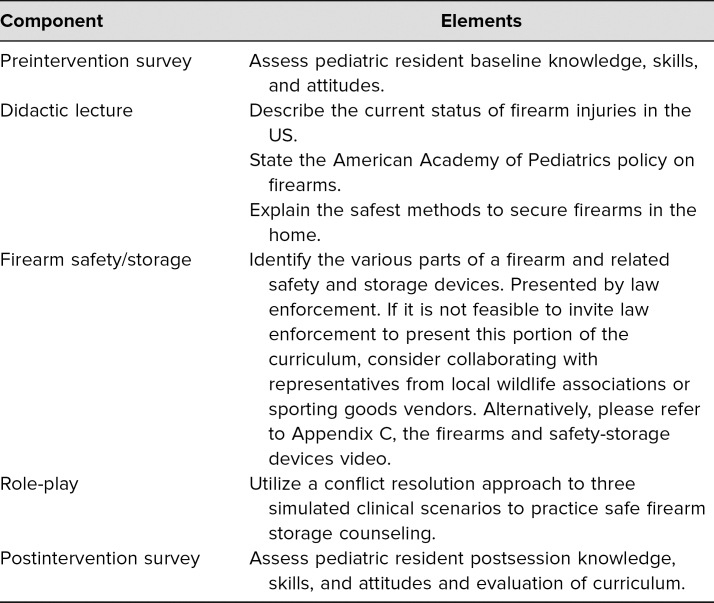
Locked and (Un-)Loaded Discussions Curriculum Design

If residency programs wish to implement this curriculum within a 60-minute teaching session, we suggest asking residents to complete the preintervention survey and review the didactic lecture and firearm and safety and storage devices video prior to the teaching session, thus allowing for sufficient time to engage in the interactive role-playing scenarios.

### Survey Distribution and Data Collection

Pre- and postintervention surveys were distributed via a QR code to a REDCap survey. REDCap was used to collect and analyze data. Surveys were anonymous, and data were deidentified. The firearm safety discussion documentation was collected via a retrospective review of medical records prior to the intervention, during a 4-week period immediately following the intervention, and during a 4-week period 12 weeks after the intervention. For programs interested in collecting firearm safety discussion electronic health record (EHR) documentation data, we have included our EHR chart audit tool (Appendix H). A consent letter was presented to residents at the beginning of the teaching session, and they were given the opportunity to opt out. The study was approved by the Baylor College of Medicine Institutional Review Board. No financial incentives were provided for participation in the study.

## Results

A total of 53 residents participated in curriculum implementation, with 52 preintervention and 48 postintervention surveys available for analysis.

**Figure 1.**
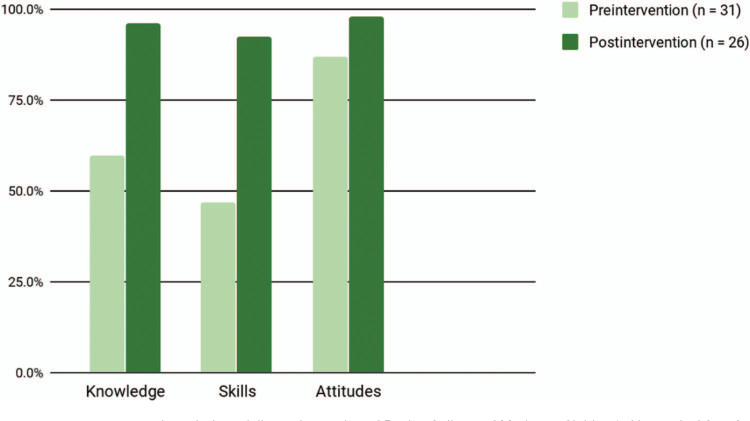
Preintervention versus postintervention knowledge, skills, and attitudes of Baylor College of Medicine-Children's Hospital of San Antonio pediatric residents.

**Figure 2.**
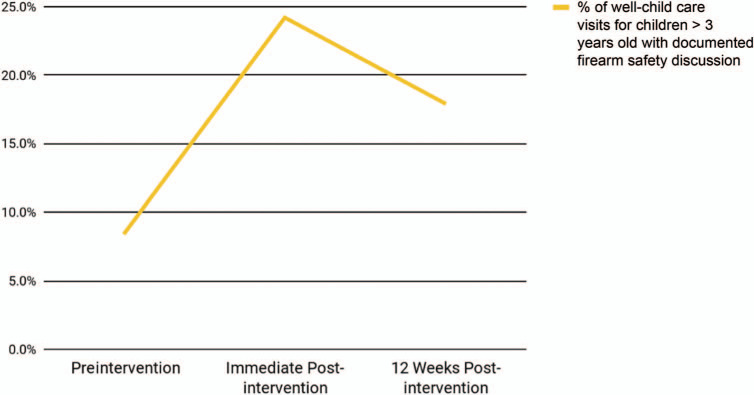
Baylor College of Medicine-Children's Hospital of San Antonio pediatric residents’ safe firearm storage counseling practices at preintervention, immediate postintervention, and 12 weeks postintervention, as documented in electronic health records during well-child care visits for children greater than 3 years old.

In the first iteration, residents completed 31 preintervention and 26 postintervention surveys. As compared to prior to the didactic session, residents reported improvement in knowledge (60% vs. 96%, *p* < .01) and skills (47% vs. 92%, *p* < .01) regarding safe firearm storage discussions with families (Figure 1). As resident preintervention attitudes were quite positive, attitudes did improve postintervention but were not statistically significant (87% vs. 98%, *p* = .13). Although 26% of residents reported often or always discussing safe firearm storage with families on the preintervention survey, a review of the EHRs for well-child care encounters with children greater than 3 years old revealed resident documentation of firearm safety discussions during roughly 8% of visits (*n* = 308) prior to the intervention, increasing to 24% (*n* = 132) and 18% (*n* = 123) immediately and 12 weeks following the intervention, respectively (*p* < .01; Figure 2). Resident evaluation comments were overwhelmingly positive. When evaluating the curriculum design, 100% of residents stated the curriculum was somewhat or very effective in achieving the educational objectives, 100% stated the format was somewhat or very effective in achieving the educational objectives, 96% stated the curriculum was somewhat or very enjoyable, and 96% stated they were somewhat or very likely to make a change in practice.

**Figure 3.**
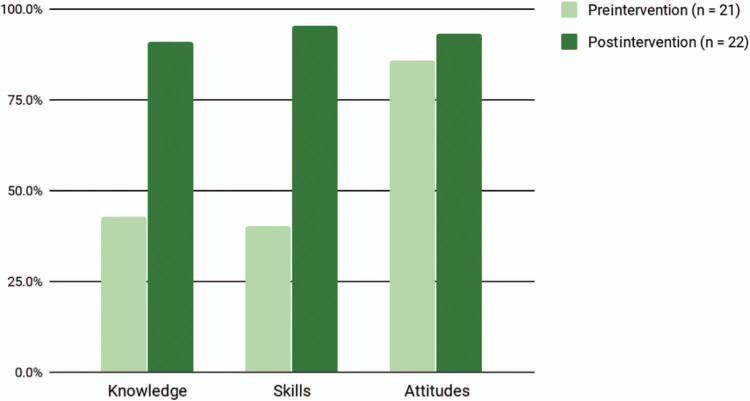
Preintervention versus postintervention knowledge, skills, and attitudes of Brooke Army Medical Center pediatric residents.

In the second iteration, 21 preintervention and 22 postintervention surveys were available for analysis. Residents again reported improvement in knowledge (43% vs. 91%, *p* < .01), skills (40% vs. 96%, *p* < .01), and attitudes (86% vs. 93%, *p* < .2; Figure 3) regarding safe firearm storage counseling following the intervention. We were unable to assess a change in behavior among this resident population via EHR documentation as we did not have access to the institution's medical records. Residents’ free-text responses upon curriculum evaluation were again vastly positive, particularly regarding strengths of the curriculum format and learning objectives that would impact their future practice. When evaluating the curriculum design, 96% of residents stated the curriculum was somewhat or very effective in achieving the educational objectives, 100% stated the format was somewhat or very effective in achieving the educational objectives, 91% stated the curriculum was somewhat or very enjoyable, and 96% stated they were somewhat or very likely to make a change in practice.

## Discussion

We designed a pediatric resident safe firearm storage counseling curriculum incorporating didactics, hands-on demonstration, and role-playing methods and implemented it within two residency programs. To our knowledge, no similar curriculum utilizing diverse teaching methods is currently available in the literature. We demonstrated a statistically significant improvement in resident knowledge and skills (Kirkpatrick hierarchy level 2) and, following one iteration, an immediate and delayed statistically significant change in resident behavior (Kirkpatrick hierarchy level 3). Residents perceived the curriculum and its format to be effective in achieving the educational objectives and enjoyed the experience overall (Kirkpatrick hierarchy level 1).

Both peer-reviewed literature^8,9^ and our own local needs assessment collected via preintervention surveys demonstrate the lack of pediatric resident comfort when providing safe firearm storage anticipatory guidance to parents/caregivers. This curriculum not only illustrated the ability to ensure that residents were equipped with the AAP recommendations on firearms in the home but also established a relationship with law enforcement, allowing for a hands-on component that developed residents’ skills in conducting these conversations. Furthermore, residents expressed an appreciation for the format of the curriculum. Specifically, responses to postintervention surveys revealed the educational value of the law enforcement presentation of firearms and firearm safety and storage devices as well as the safe opportunity to role-play counseling discussions during the interactive scenarios.

Postintervention survey data following the first implementation of the curriculum informed modifications of our methods. Notably, investigators observed that assembling small groups of five to six residents was not conducive to active participation by all group members. During the second iteration, residents were asked to form groups of three, wherein one resident assumed the role of parent, one assumed the role of pediatrician, and the third observed the interaction and provided feedback. This arrangement permitted equal active participation. Because of time constraints and, to a lesser extent, the assumption that residents in military programs were well versed in this topic, the law enforcement demonstration video was not presented, and residents asserted the essential nature of this component of the curriculum. A link to the video was shared with the residency program subsequently. Although a concerted effort was made to exclude politics from the teaching session and two participants endorsed the unbiased delivery of educational material, one resident did recommend rephrasing specific statements to ensure impartiality. Educators are encouraged to assess their own biases and messaging when delivering the didactic portion of the curriculum.

Although the target learners of this curriculum are pediatric residents, our educational product is adaptable and can be implemented among practicing physicians as well. Indeed, a review of the literature reveals that posttraining, providers are also uncomfortable conducting these discussions, establishing the need for such a curriculum. Location-specific data are not included, although adult clinicians may choose to replace the child-specific statistics with those more relevant to their practice.

Limitations of this resource include its having been implemented in only two pediatric residency programs in the same city, which may limit generalizability to other settings or target learners. Furthermore, there is an inability to truly assess a change in resident behavior unless this information is readily retrievable from the EHR. Finally, we reviewed medical records of encounters occurring within 4 months of delivering the curriculum, and it is unclear if this educational resource has had a sustained impact on behavior.

This curriculum assembles diverse teaching methods to improve pediatric resident knowledge, skills, attitudes, and, ultimately, behavior when initiating safe firearm storage counseling discussions with parents/caregivers. Safe firearm storage in children's homes is critical to preventing unintentional pediatric firearm injuries.

## Appendices

Preintervention Survey.docxDidactic Lecture.pptxFirearm & Safety-Storage Devices.mp4Sample Phone Script & Email to Law Enforcement.docxRole-Playing Scenarios.docxFacilitators Guide for Role-Playing Scenarios.docxPostintervention Survey.docxEHR Chart Audit Tool.docx
All appendices are peer reviewed as integral parts of the Original Publication.

## References

[1] Council on Injury, Violence, and Poison Prevention Executive Committee. Firearm-related injuries affecting the pediatric population. Pediatrics. 2012;130(5):e1416–e1423. 10.1542/peds.2012-2481 23080412

[2] National Center for Health Statistics. Adolescent health. Centers for Disease Control and Prevention. Accessed April 11, 2020. https://www.cdc.gov/nchs/fastats/adolescent-health.htm

[3] Miller M, Hemenway D The relationship between firearms and suicide: a review of the literature. Aggress Violent Behav. 1999;4(1):59–75. 10.1016/S1359-1789(97)00057-8

[4] Angelmyer A, Horvath T, Rutherford G The accessibility of firearms and risk for suicide and homicide victimization among household members: a systematic review and meta-analysis. Ann Intern Med. 2014;160(2):101–110. 10.7326/M13-1301 24592495

[5] Betz ME, Barber C, Miller M Suicidal behavior and firearm access: results from the Second Injury Control and Risk Survey. Suicide Life Threat Behav. 2011;41(4):384–391. 10.1111/j.1943-278X.2011.00036.x 21535097

[6] Gun Violence Archive 2020. Gun Violence Archive. Updated April 9, 2020. Accessed April 11, 2020. https://www.gunviolencearchive.org/

[7] Naik-Mathuria B, Gill AC Firearm injuries in children: prevention. UpToDate. Updated February 7, 2020. Accessed April 11, 2020. https://www.uptodate.com/contents/firearm-injuries-in-children-prevention

[8] Price JH, Thompson A, Khubchandani J, Wiblishauser M, Dowling J, Teeple K Perceived roles of emergency department physicians regarding anticipatory guidance on firearm safety. J Emerg Med. 2013;44(5):1007–1016. 10.1016/j.jemermed.2012.11.010 23352862

[9] Solomon BS, Duggan AK, Webster D, Serwint JR Pediatric residents’ attitudes and behaviors related to counseling adolescents and their parents about firearm safety. Arch Pediatr Adolesc Med. 2002;156(8):769–775. 10.1001/archpedi.156.8.769 12144366

[10] Juang DD, McDonald DL, Johnson-Young EA, et al Assessment of pediatric residents’ attitudes toward anticipatory counseling on gun safety. Children. 2019;6(11):122 10.3390/children6110122 PMC6915471 31683753

[11] Butkus R, Weissman A Internists’ attitudes toward prevention of firearm injury. Ann Intern Med. 2014;160(12):821–827. 10.7326/M13-1960 24722784

[12] Betz ME, Azrael D, Barber C, Miller M Public opinion regarding whether speaking with patients about firearms is appropriate: results of a national survey. Ann Intern Med. 2016;165(8):543–550. 10.7326/M16-073913. 27455516

[13] With millions of kids at home, we must make sure firearms are stored securely. Be SMART. Accessed April 11, 2020. http://besmartforkids.org/.

[14] Wolfe AD, Hoang KB, Denniston SF Teaching conflict resolution in medicine: lessons from business, diplomacy, and theatre. MedEdPORTAL. 2018;14:10672 10.15766/mep_2374-8265.10672. 30800872 PMC6342419

