# Supplementary material for: Locked and (Un)-Loaded Discussions: A Pediatric Resident Safe Firearm Storage Counseling Curriculum
Source: MedEdPORTAL. 2020 Dec 4;16:11028. doi: 10.15766/mep_2374-8265.11028 (PMC7727610; doi:10.15766/mep_2374-8265.11028)
Supplement: Supplementary file 1 — Preintervention Survey.docxDidactic Lecture.pptxFirearm & Safety-Storage Devices.mp4Sample Phone Script & Email to Law Enforcement.docxRole-Playing Scenarios.docxFacilitators Guide for Role-Playing Scenarios.docxPostintervention Survey.docxEHR Chart Audit Tool.docx [file mep_2374-8265.11028-s001.zip › A. Preintervention Survey.docx]

**Locked and (Un)-Loaded Discussions Pre-Intervention Survey**

How familiar are you with the American Academy of Pediatrics (AAP) recommendations for safe firearm storage?

__Not familiar at all

__Somewhat familiar

__Moderately familiar

__Quite a bit familiar

__Very familiar

How important is discussing safe firearm storage with parents/caregivers?

__Not important at all

__Somewhat important

__Moderately important

__Quite a bit important

__Very important

How comfortable are you discussing safe firearm storage with parents/caregivers?

__Not comfortable at all

__Somewhat comfortable

__Moderately comfortable

__Quite a bit comfortable

__Very comfortable

How often do you discuss safe firearm storage with parents/caregivers?

__Never

__Rarely

__Sometimes

__Often

__Always

How familiar are you with the safety features of a firearm and its accessories (a safety, a gun lock, a biometric safe)?

__Not familiar at all

__Somewhat familiar

__Moderately familiar

__Quite a bit familiar

__Very familiar

How comfortable are you discussing the safety features of a firearm and its accessories (a safety, a gun lock, a biometric safe)?

__Not comfortable at all

__Somewhat comfortable

__Moderately comfortable

__Quite a bit comfortable

__Very comfortable

How important is it to know the safety features of a firearm and its accessories (a safety, a gun lock, a biometric safe) when discussing safe firearm storage with parents/caregivers?

__Not important at all

__Somewhat important

__Moderately important

__Quite a bit important

__Very important

Do you believe that safe firearm storage counseling will prevent unintentional firearm injuries/fatalities among children?

__Yes

__No
