# Supplementary material for: Locked and (Un)-Loaded Discussions: A Pediatric Resident Safe Firearm Storage Counseling Curriculum
Source: MedEdPORTAL. 2020 Dec 4;16:11028. doi: 10.15766/mep_2374-8265.11028 (PMC7727610; doi:10.15766/mep_2374-8265.11028)
Supplement: Supplementary file 1 — Preintervention Survey.docxDidactic Lecture.pptxFirearm & Safety-Storage Devices.mp4Sample Phone Script & Email to Law Enforcement.docxRole-Playing Scenarios.docxFacilitators Guide for Role-Playing Scenarios.docxPostintervention Survey.docxEHR Chart Audit Tool.docx [file mep_2374-8265.11028-s001.zip › B. Didactic Lecture.pptx]

## Slide 1
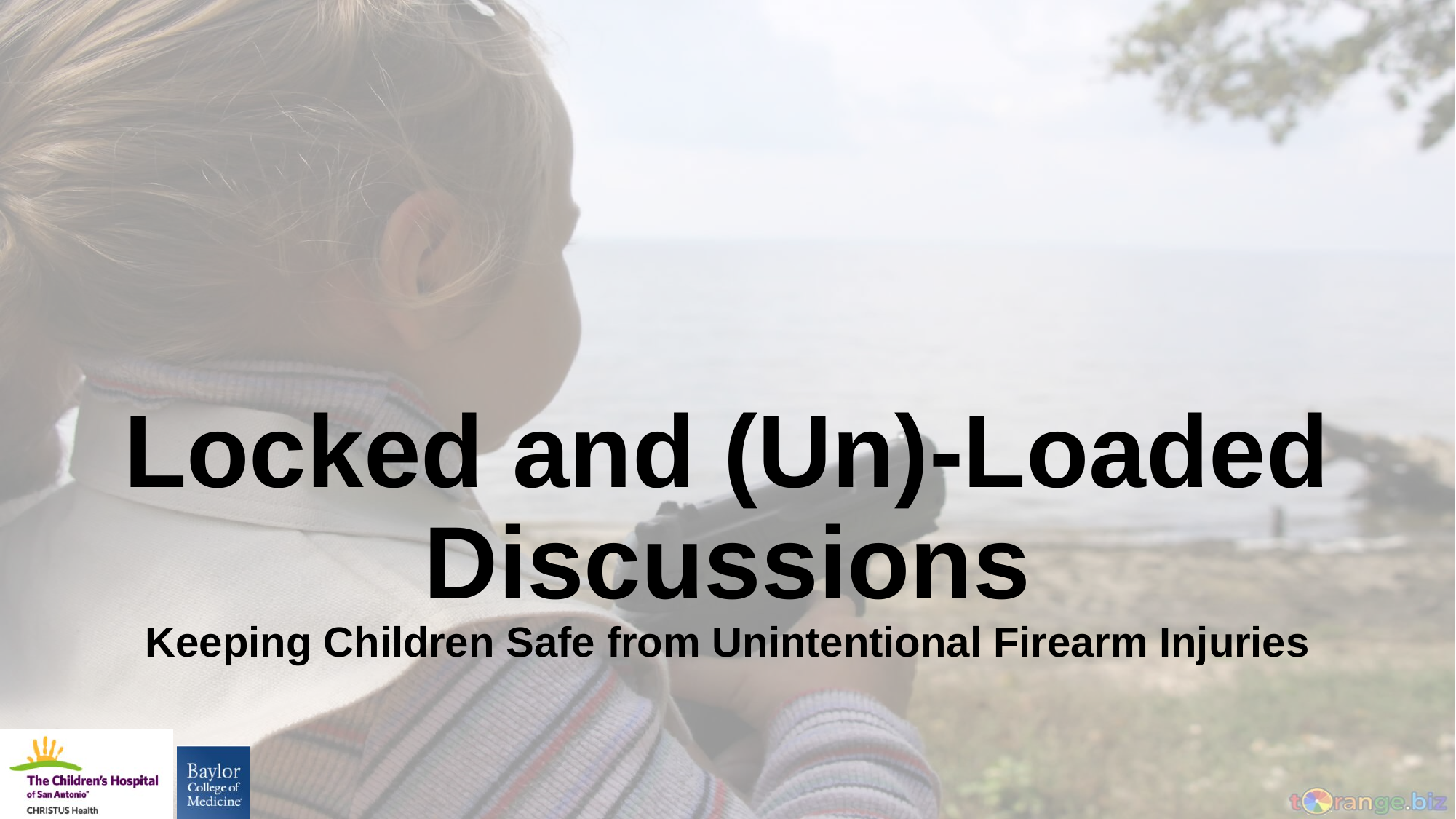

# Locked and (Un)-Loaded Discussions Keeping Children Safe from Unintentional Firearm Injuries

## Slide 2
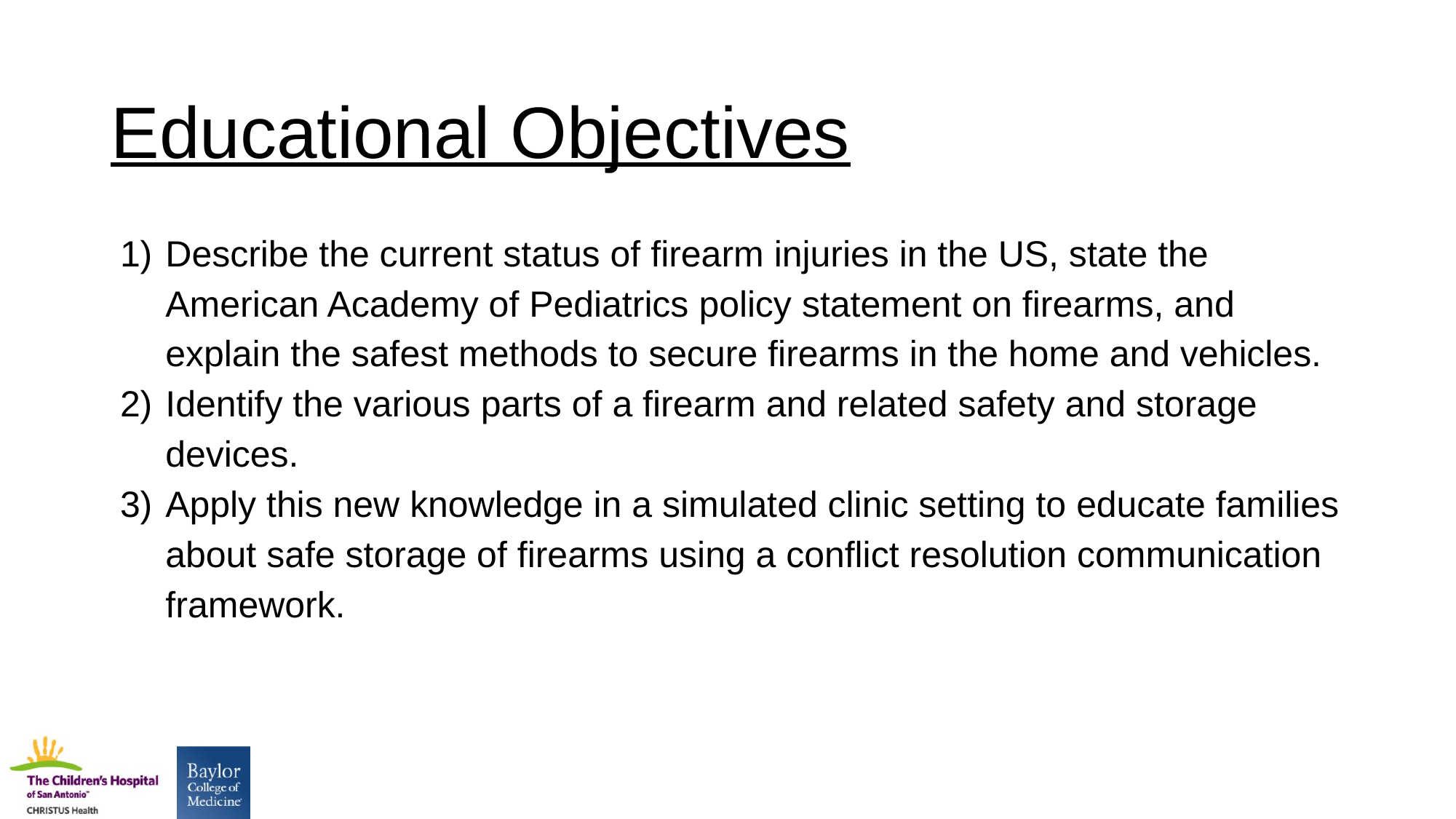

# Educational Objectives
Describe the current status of firearm injuries in the US, state the American Academy of Pediatrics policy statement on firearms, and explain the safest methods to secure firearms in the home and vehicles.
Identify the various parts of a firearm and related safety and storage devices.
Apply this new knowledge in a simulated clinic setting to educate families about safe storage of firearms using a conflict resolution communication framework.

## Slide 3
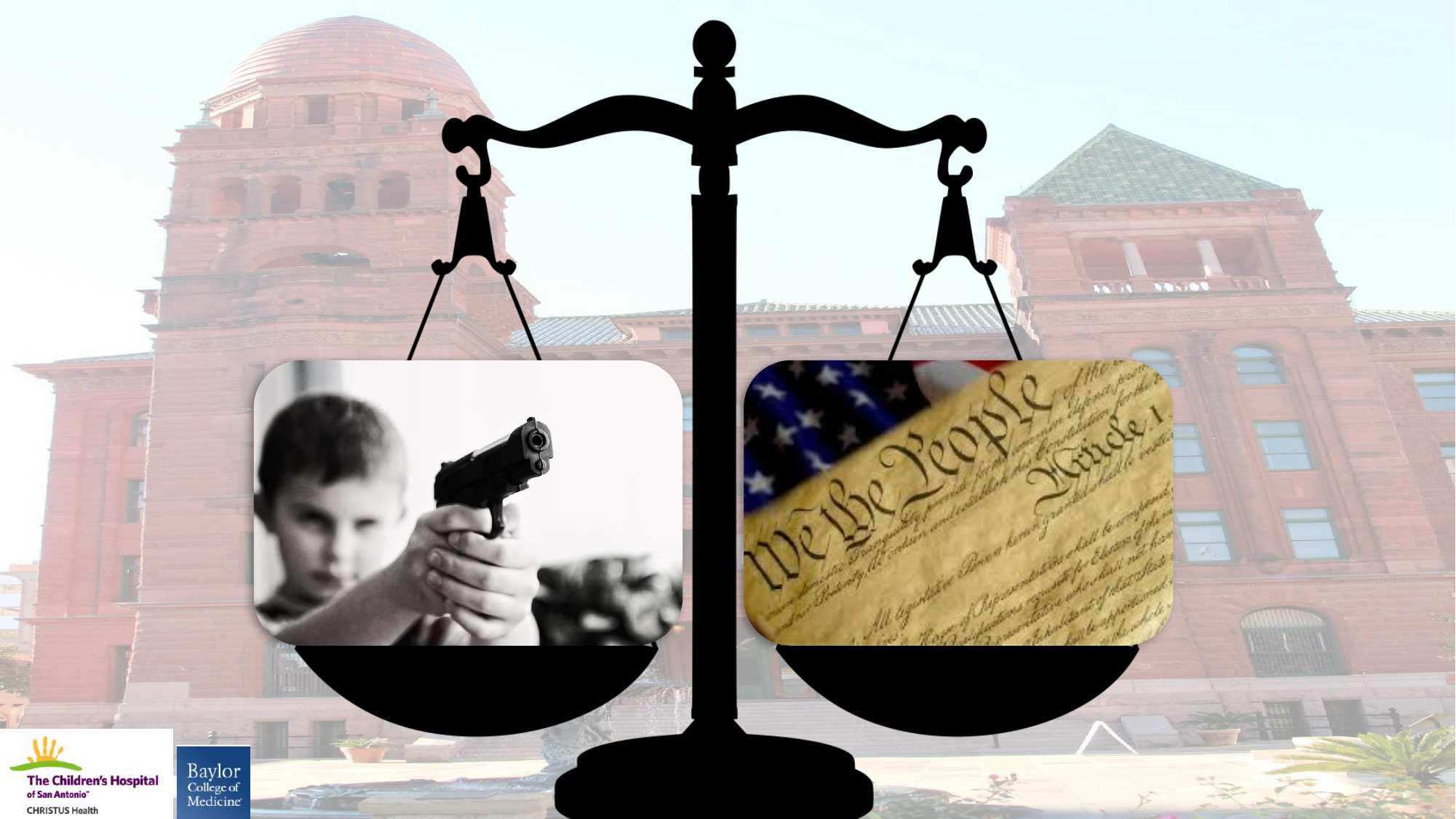

## Slide 4
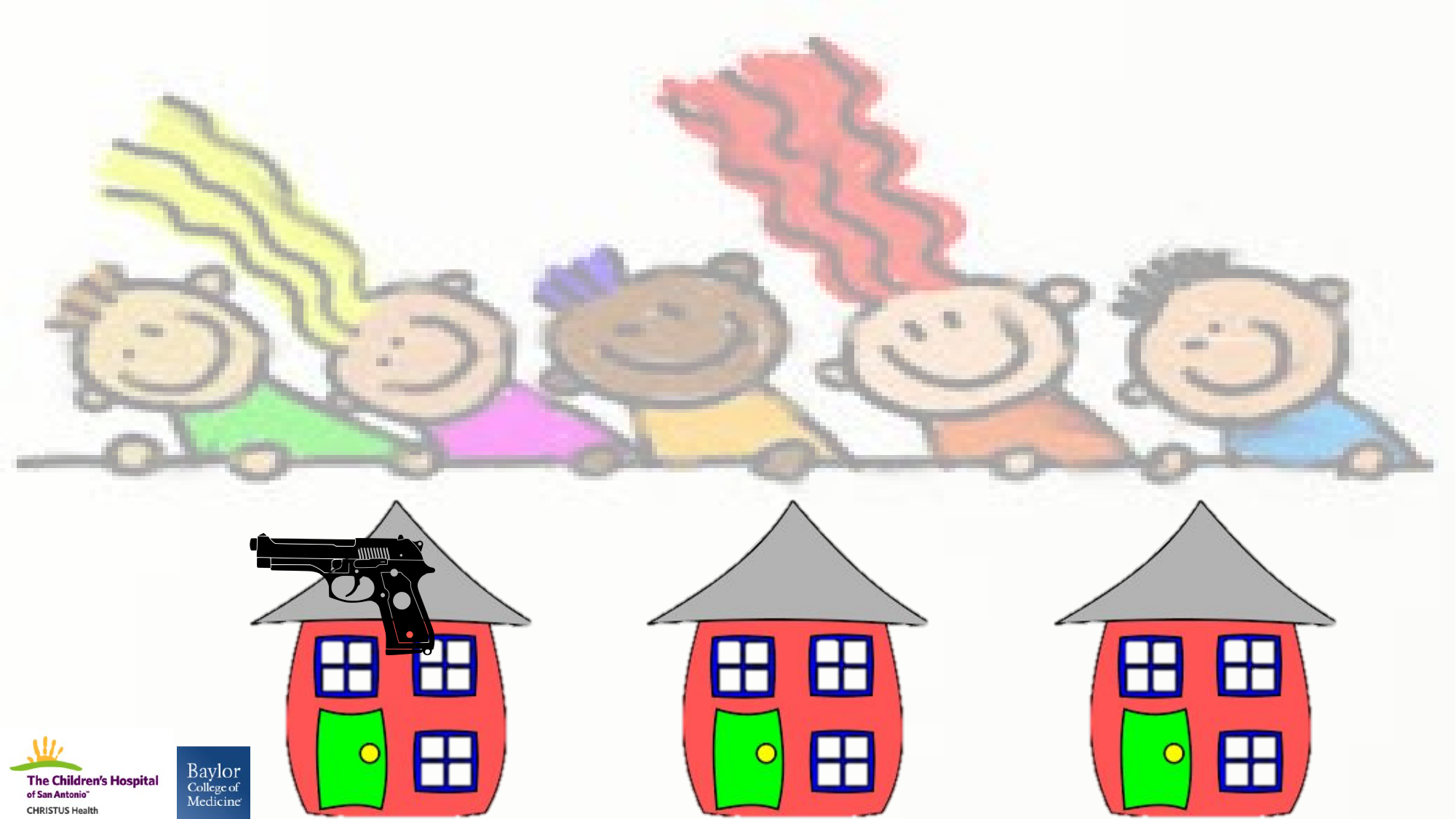

## Slide 5
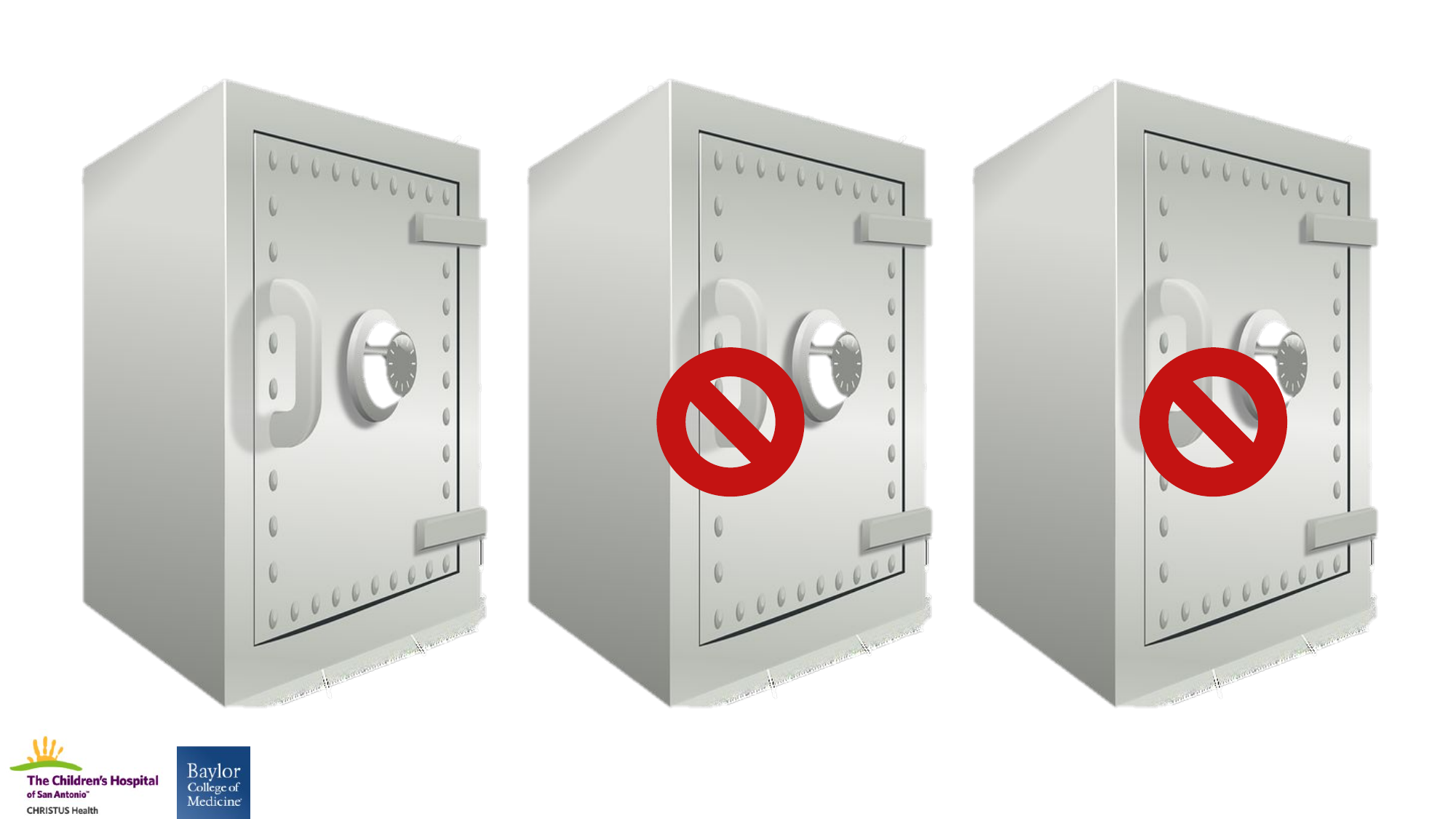

## Slide 6
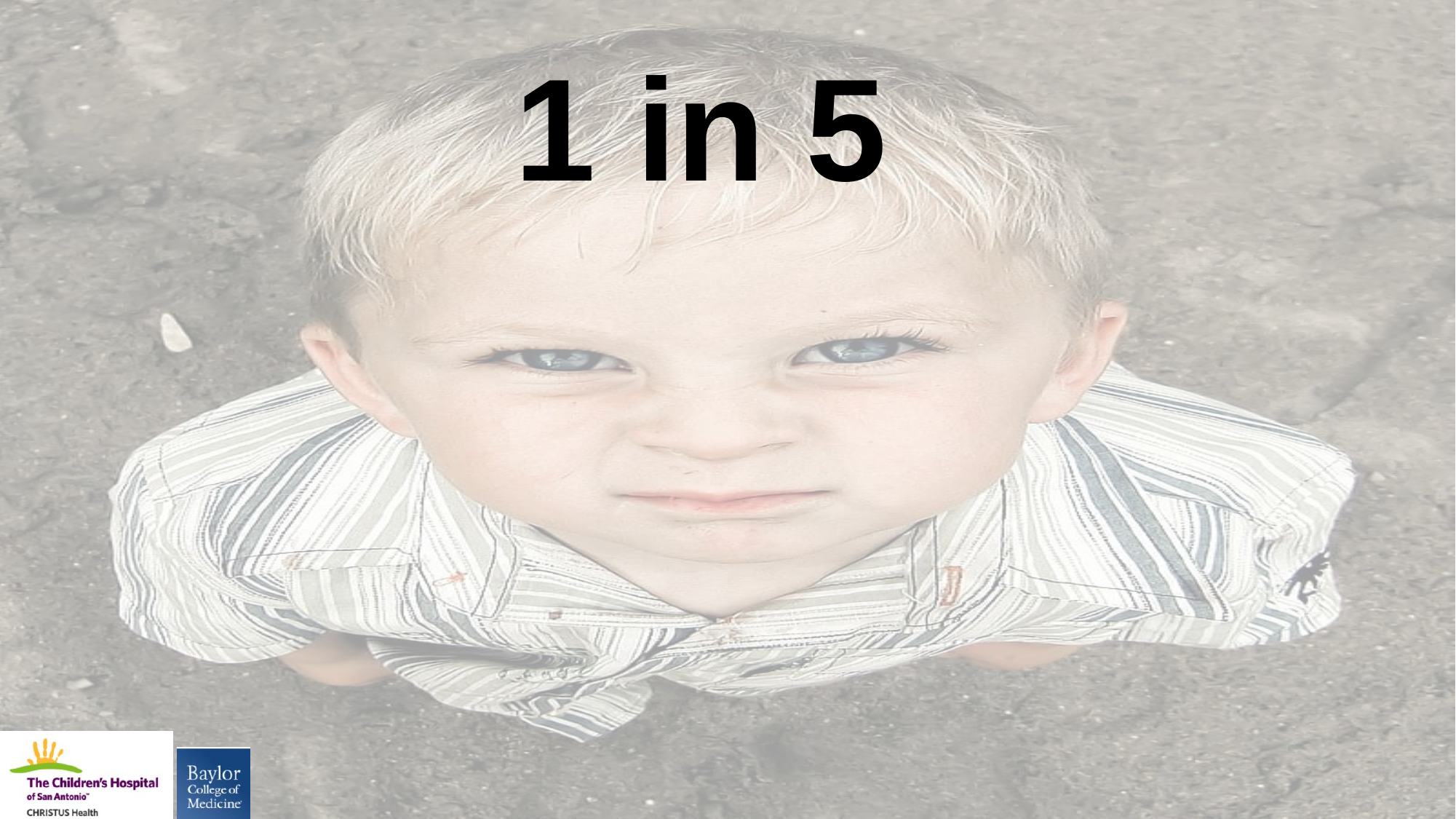

1 in 5

## Slide 7
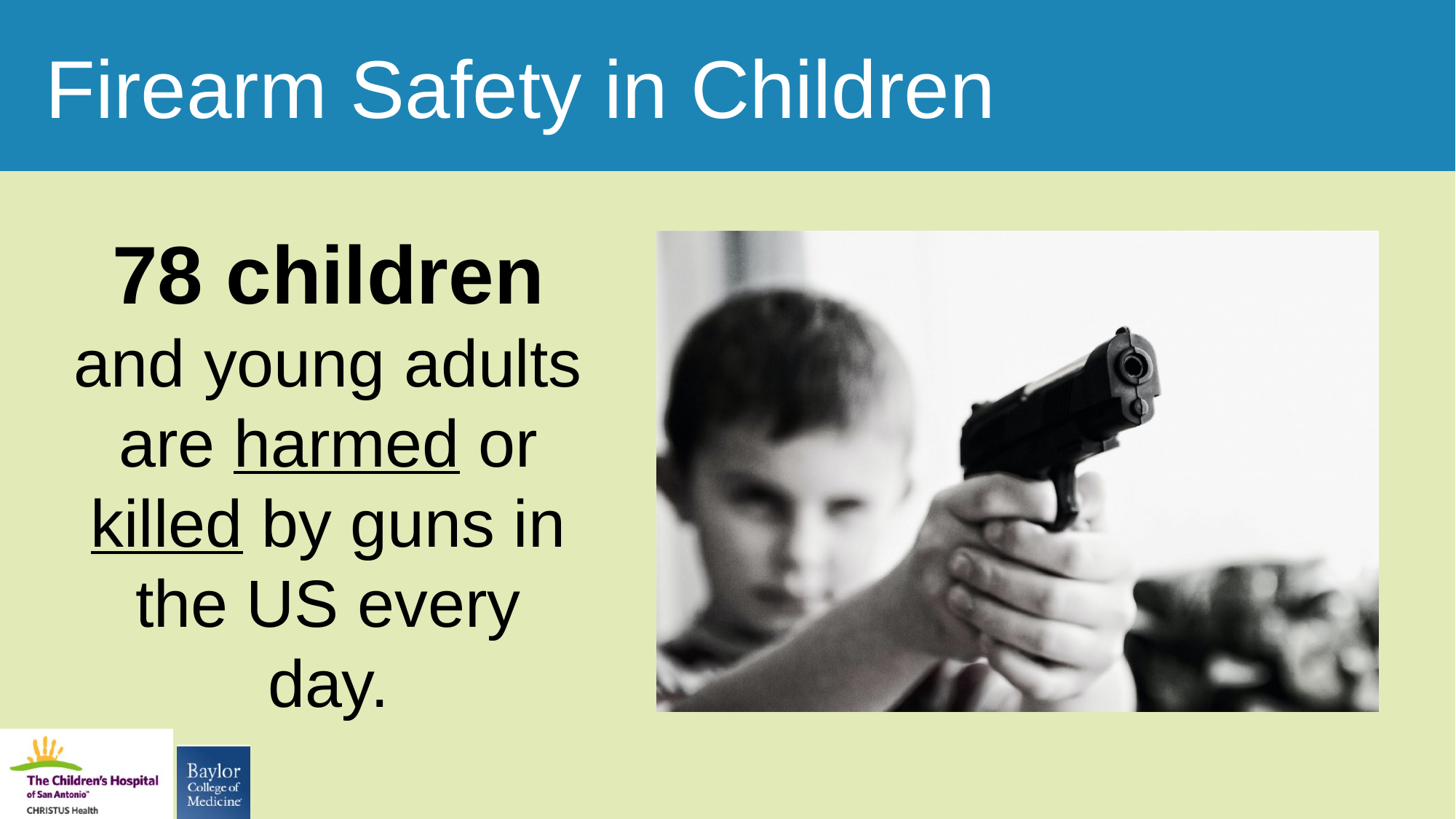

Firearm Safety in Children
78 children and young adults are harmed or killed by guns in the US every day.

## Slide 8
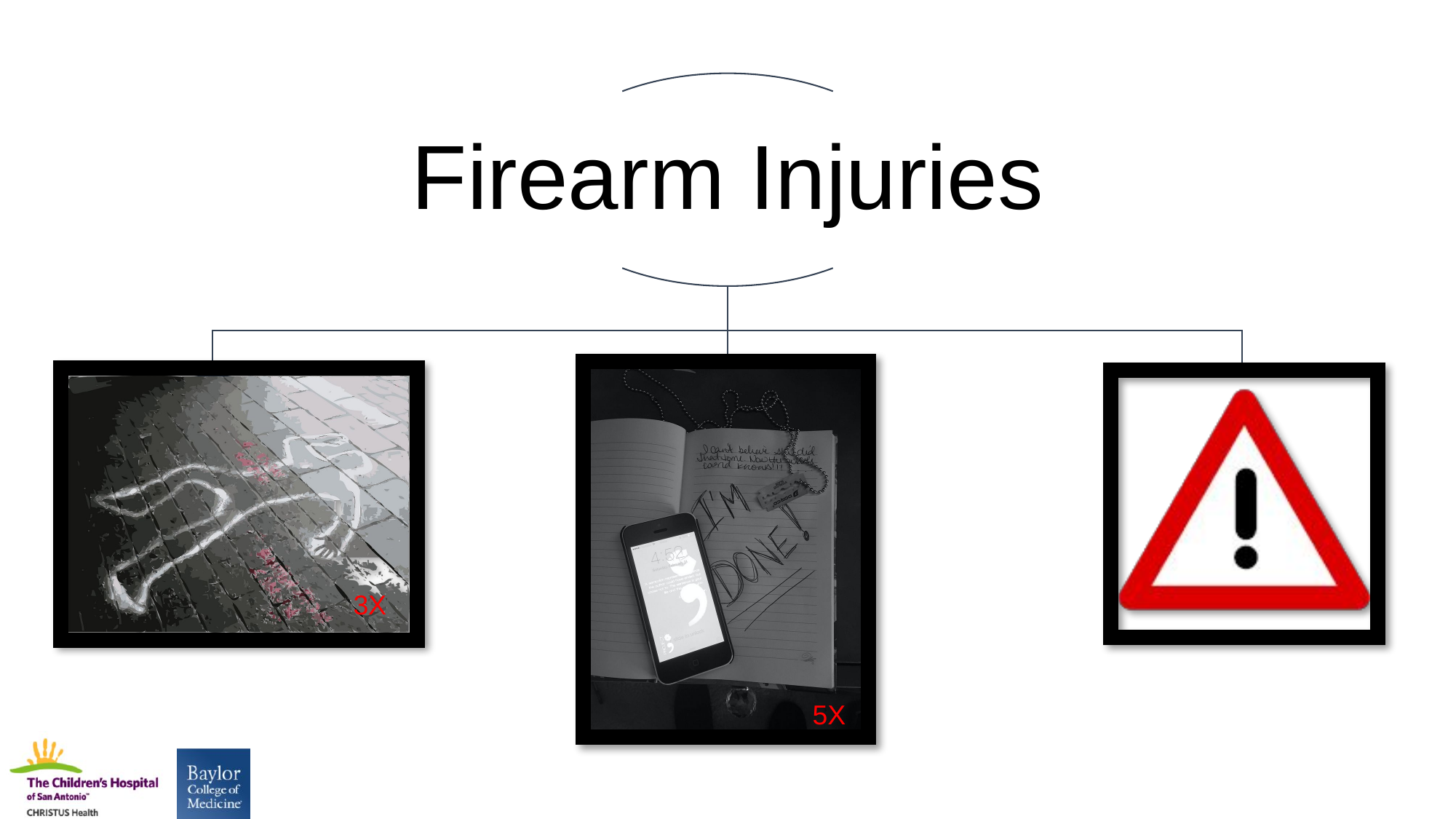

Firearm Injuries
3X
5X

## Slide 9
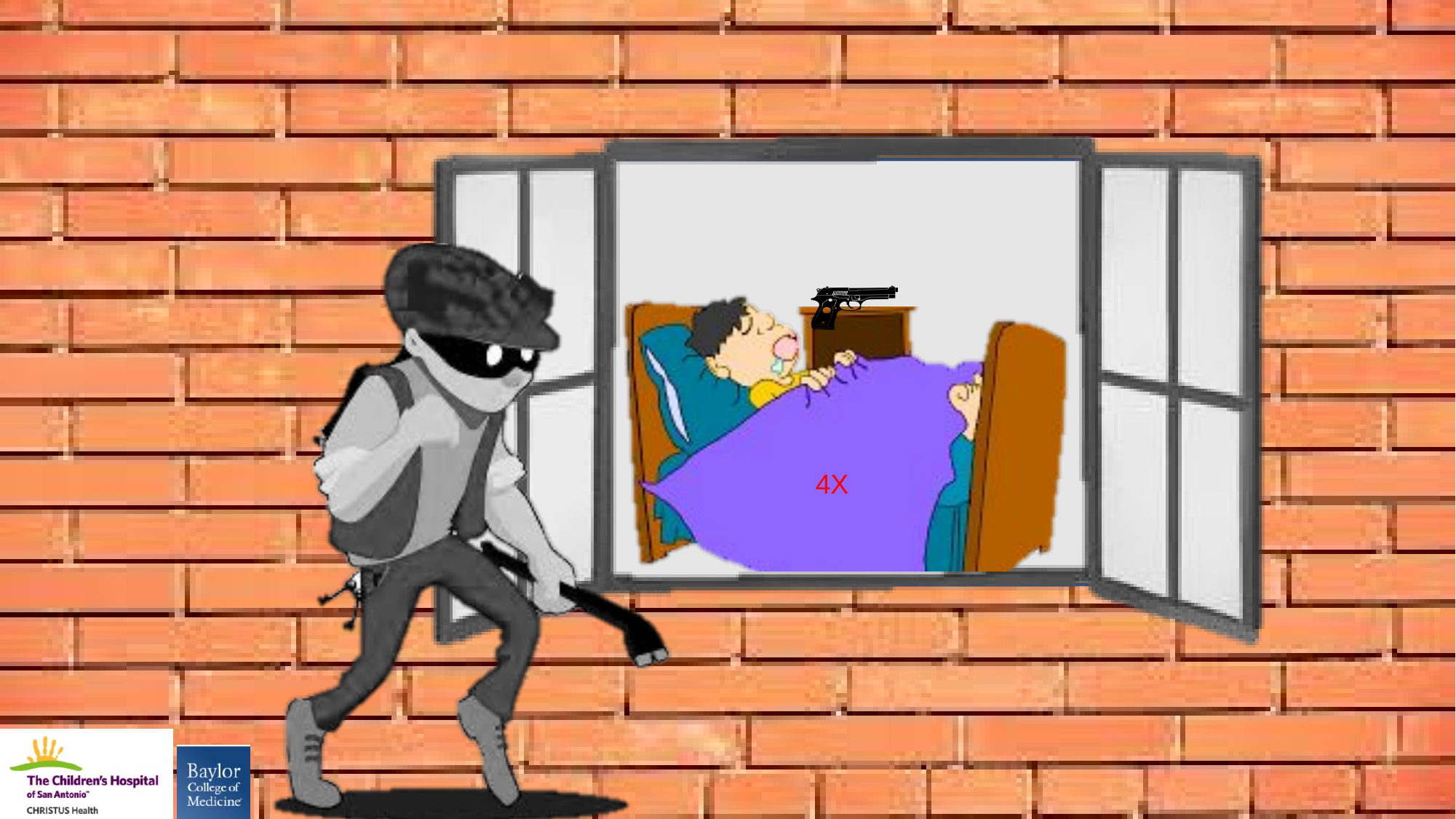

4X

## Slide 10
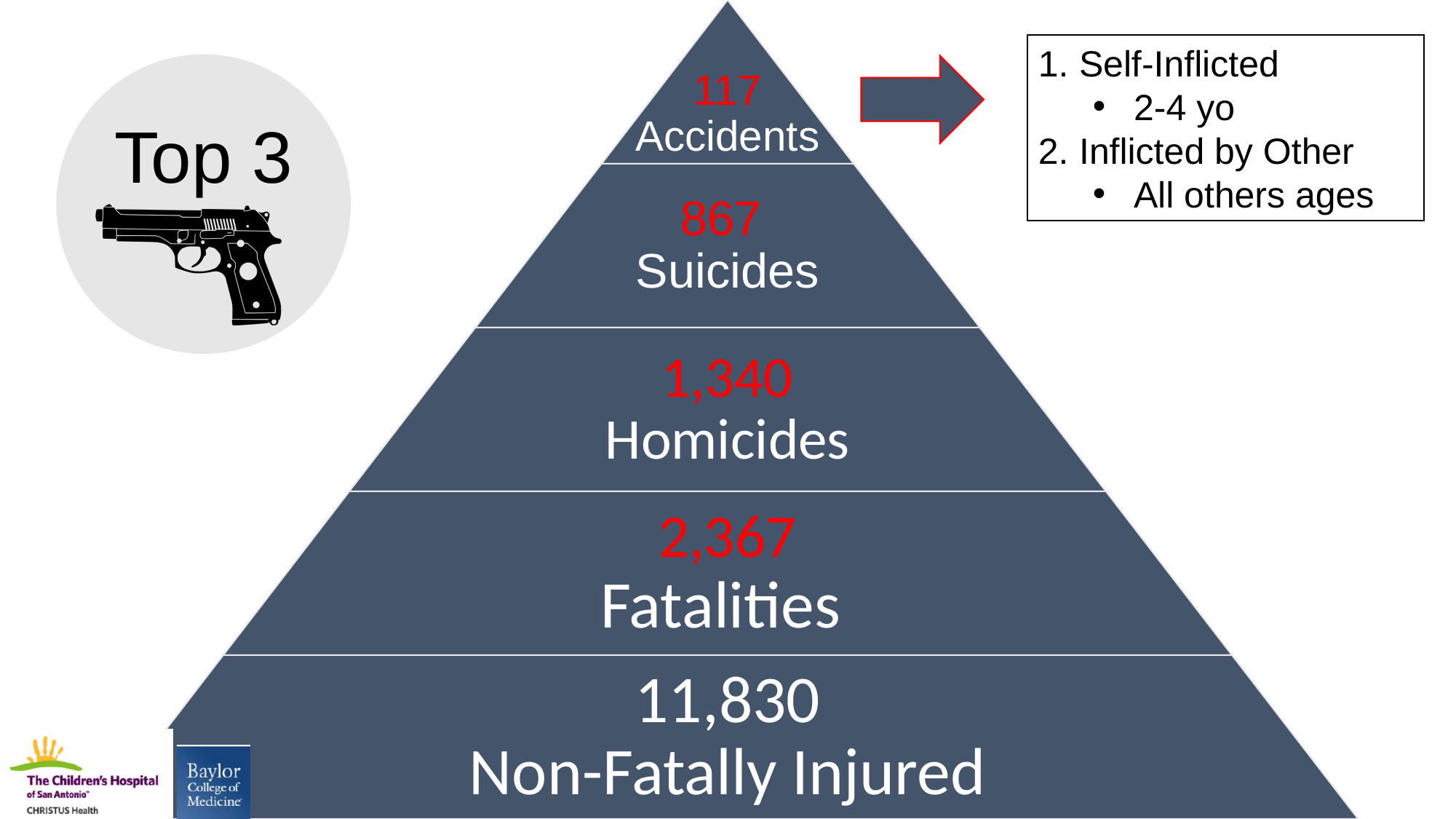

117
Accidents
867
Suicides
1,340
Homicides
2,367
Fatalities
11,830
Non-Fatally Injured
Self-Inflicted
2-4 yo
Inflicted by Other
All others ages
Top 3

## Slide 11
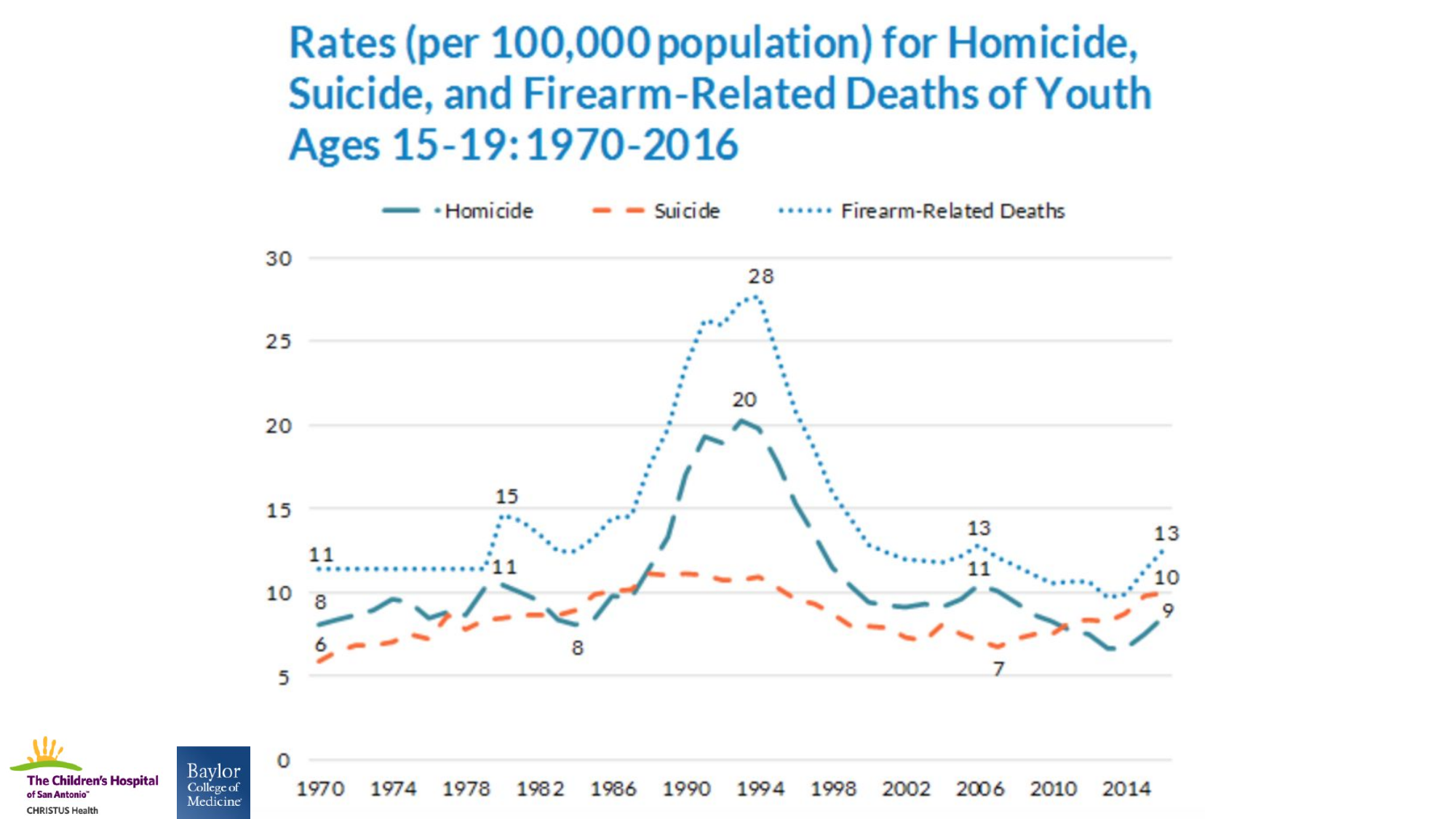

## Slide 12
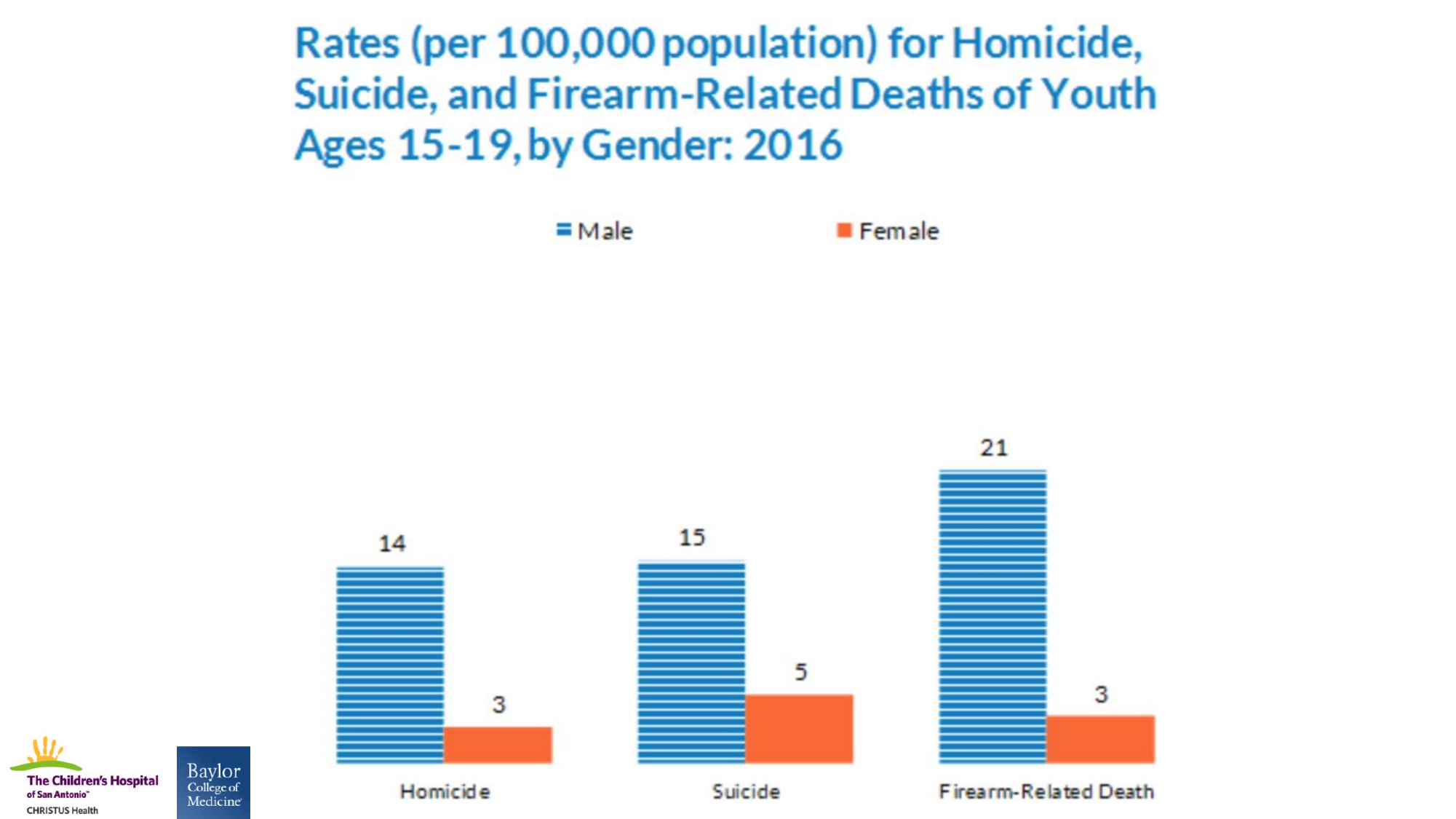

## Slide 13
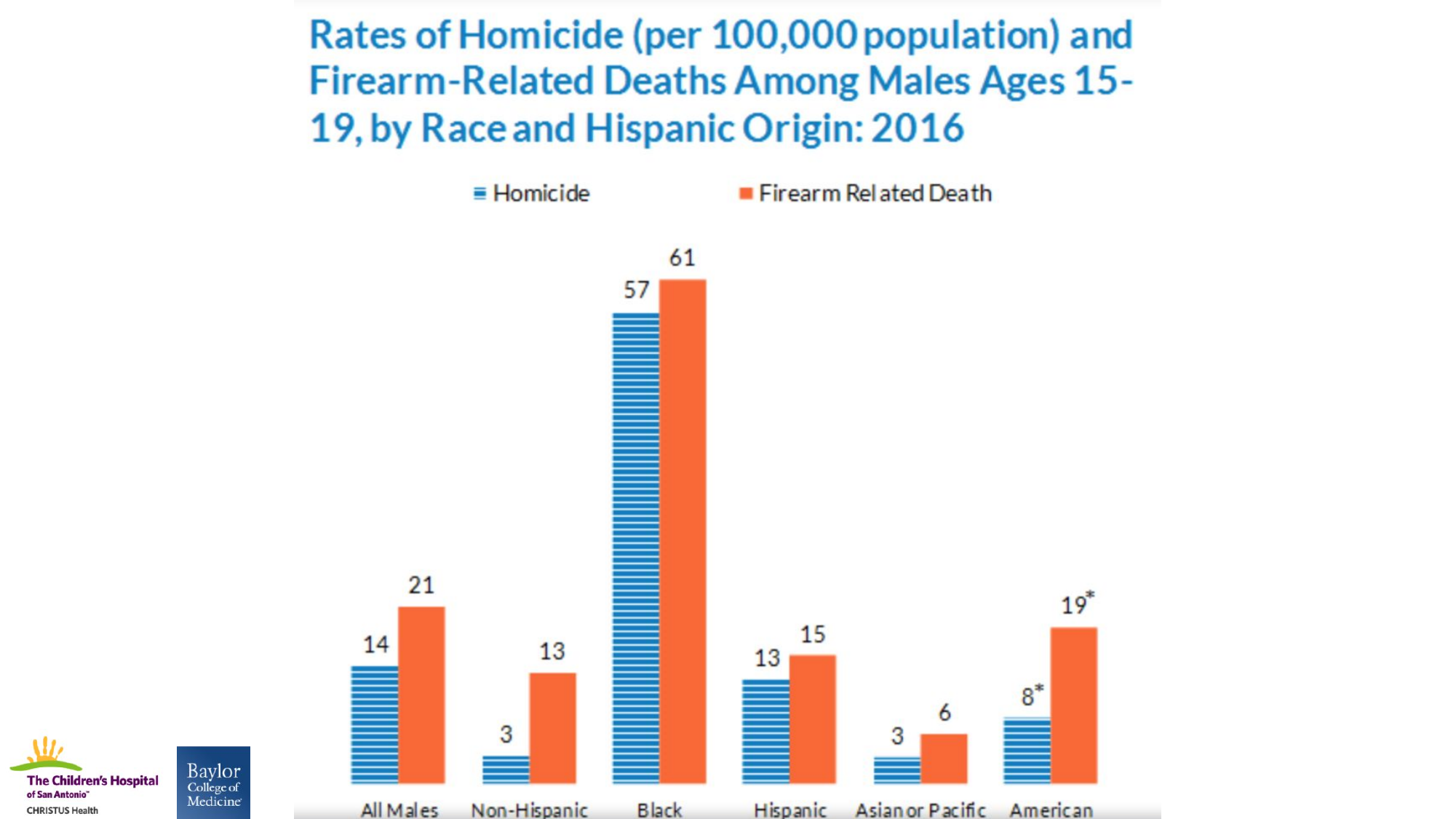

## Slide 14
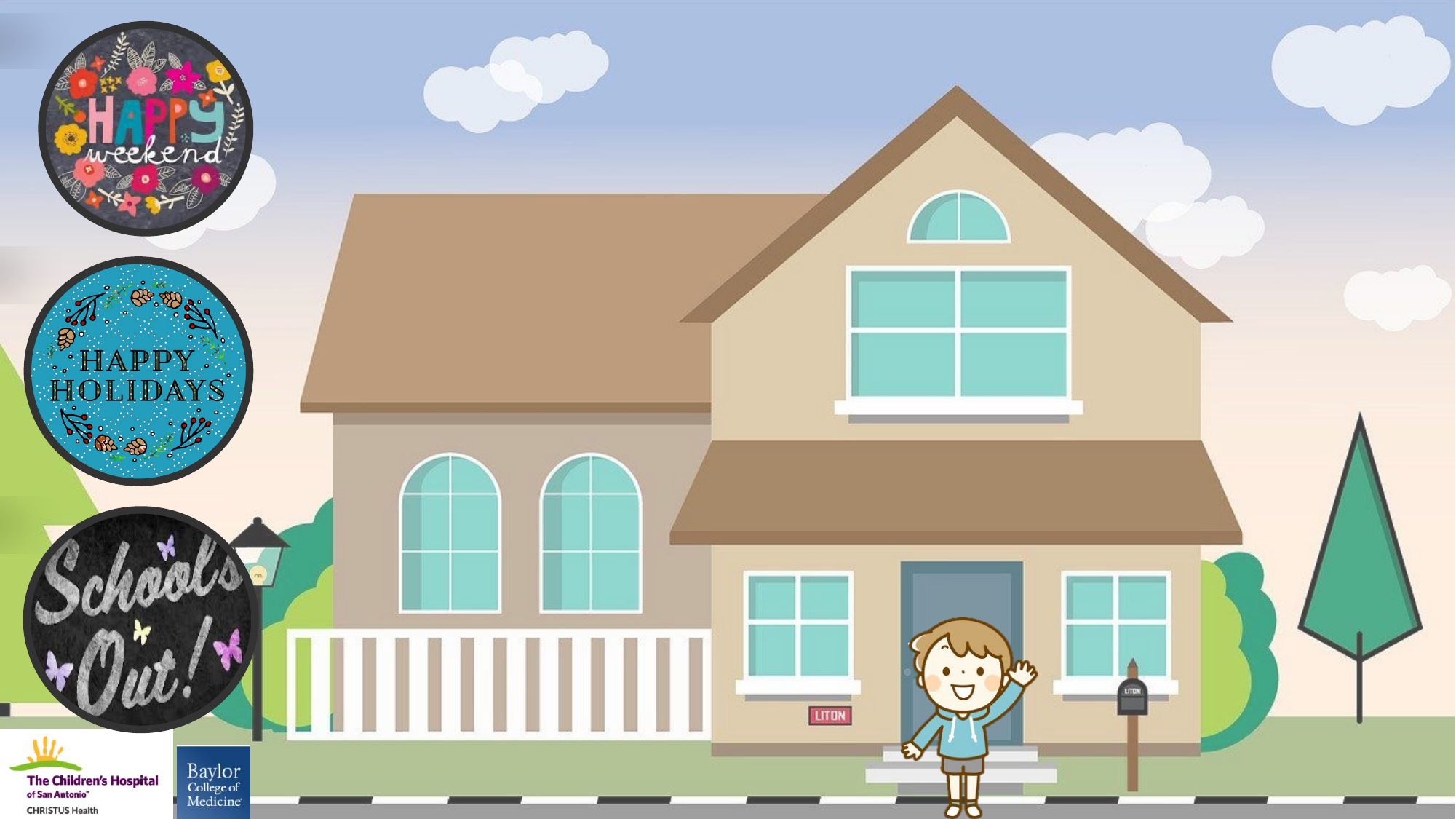

## Slide 15
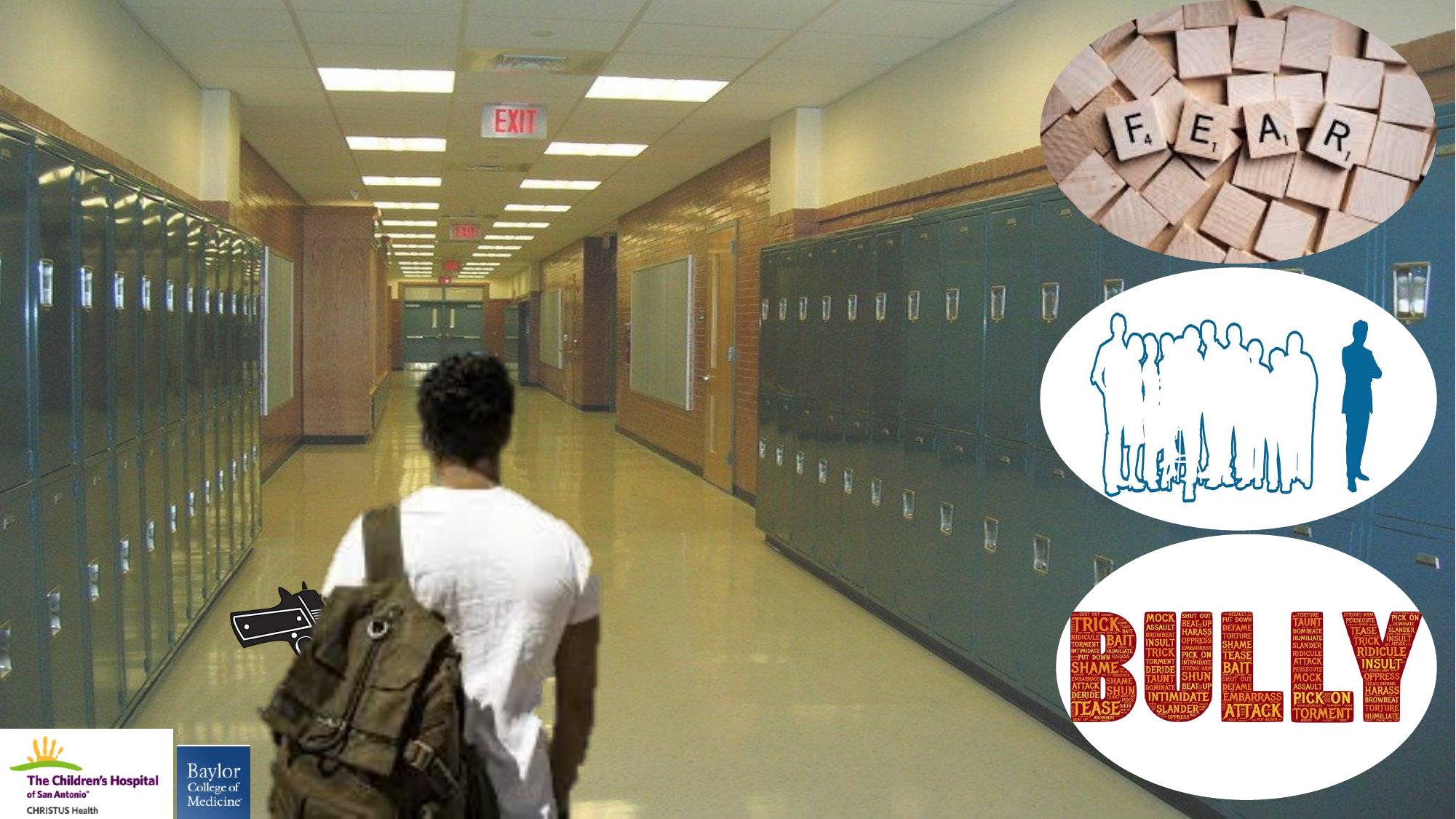

## Slide 16
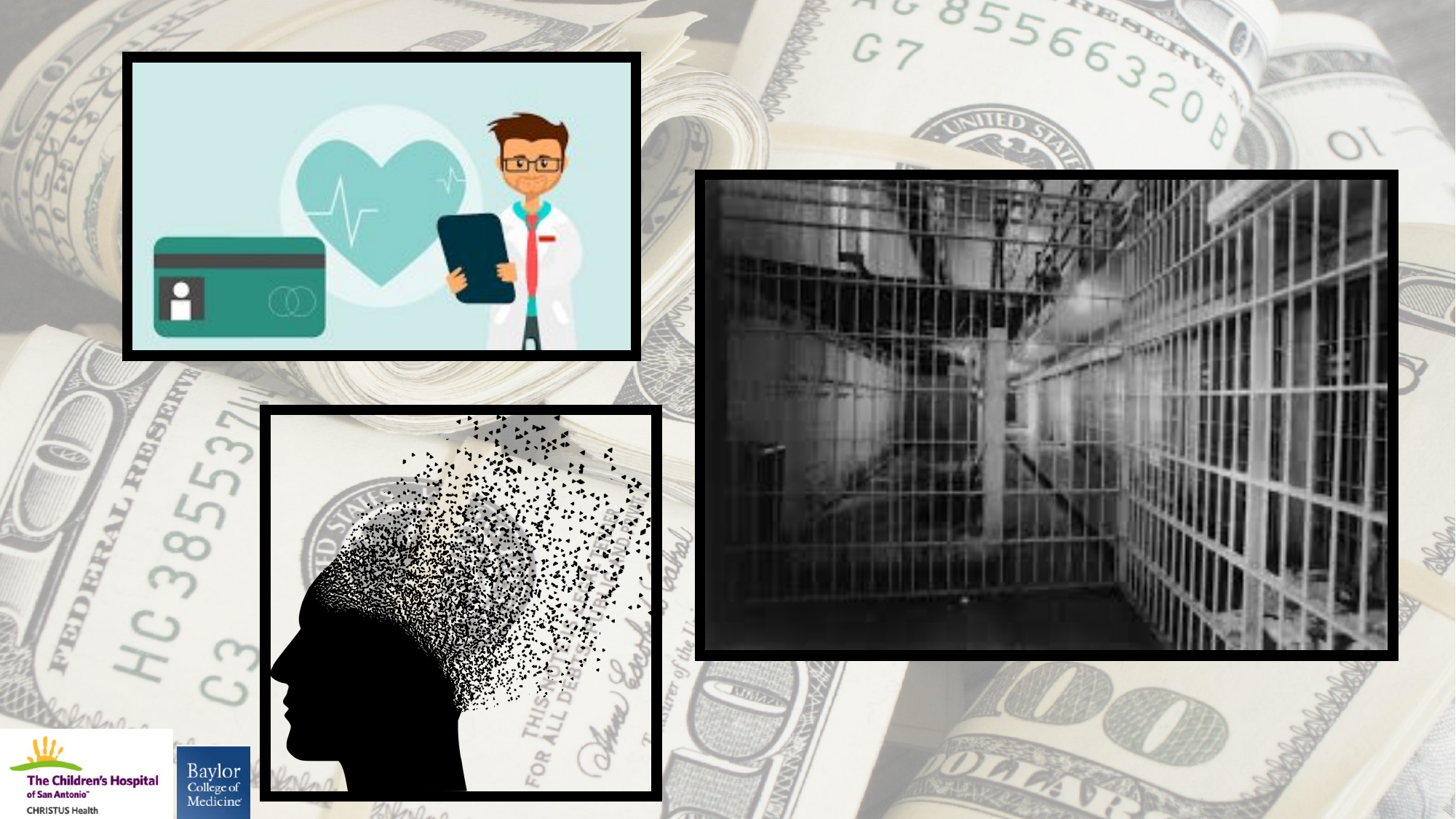

## Slide 17
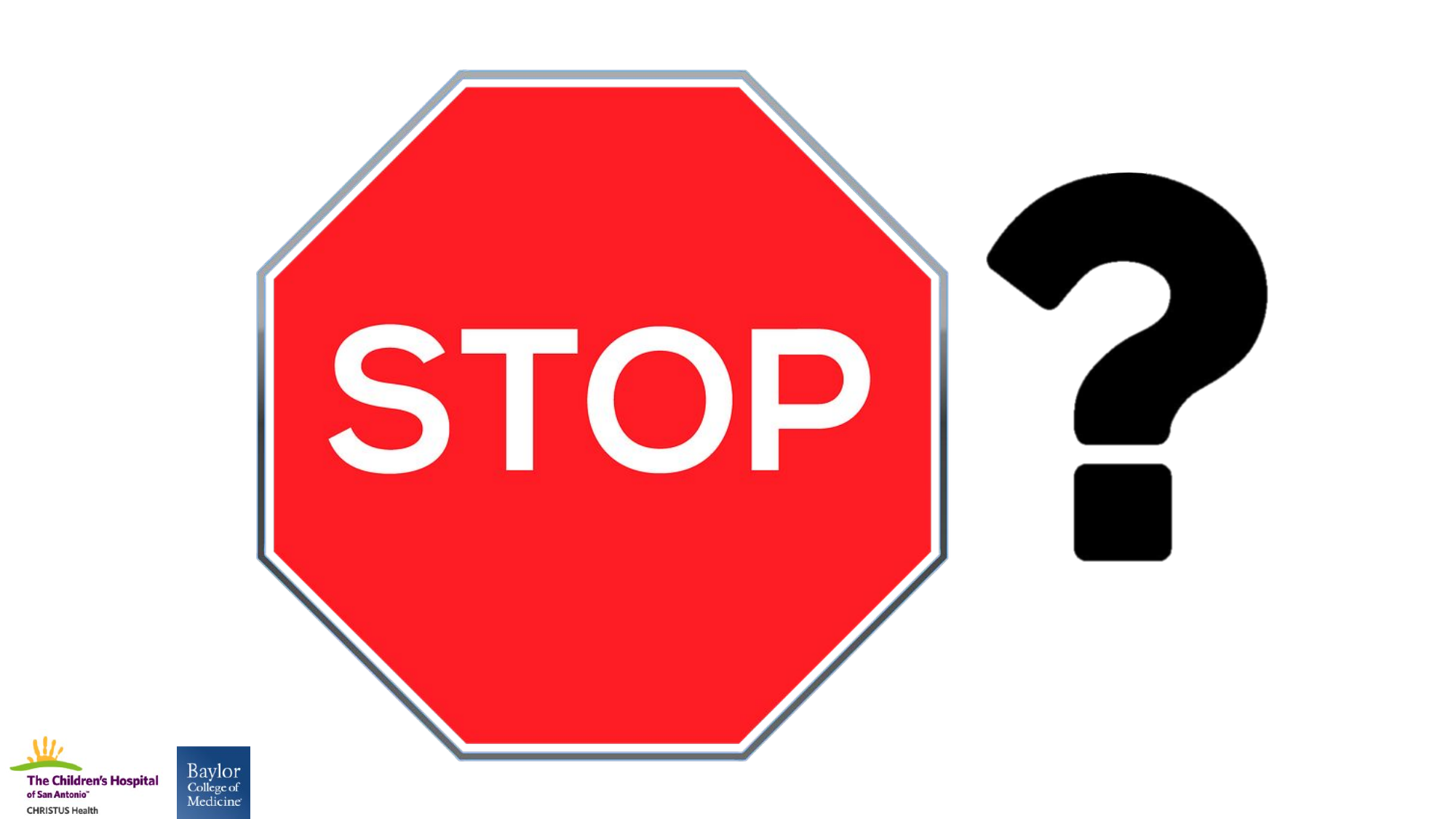

## Slide 18
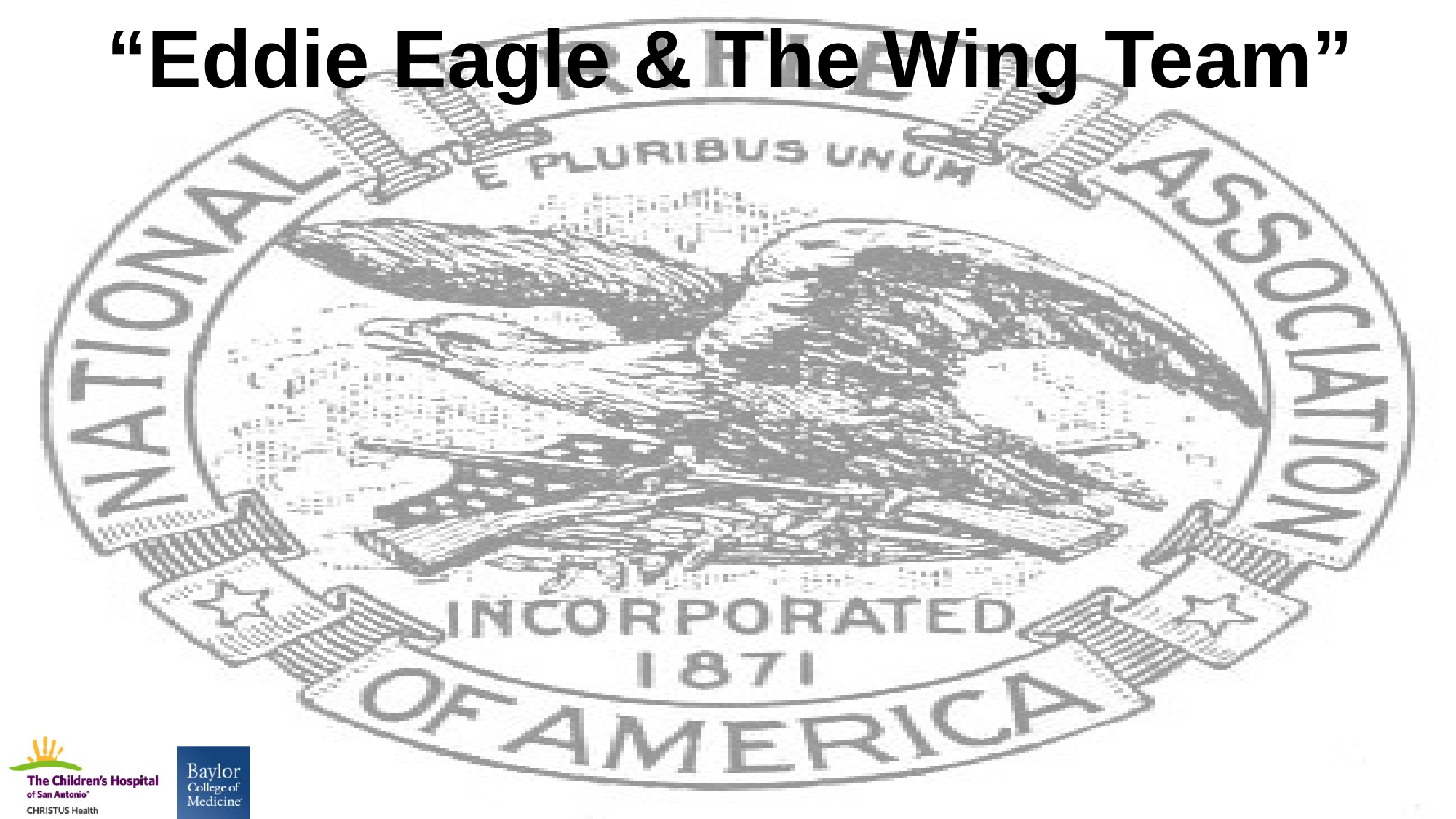

“Eddie Eagle & The Wing Team”

## Slide 19
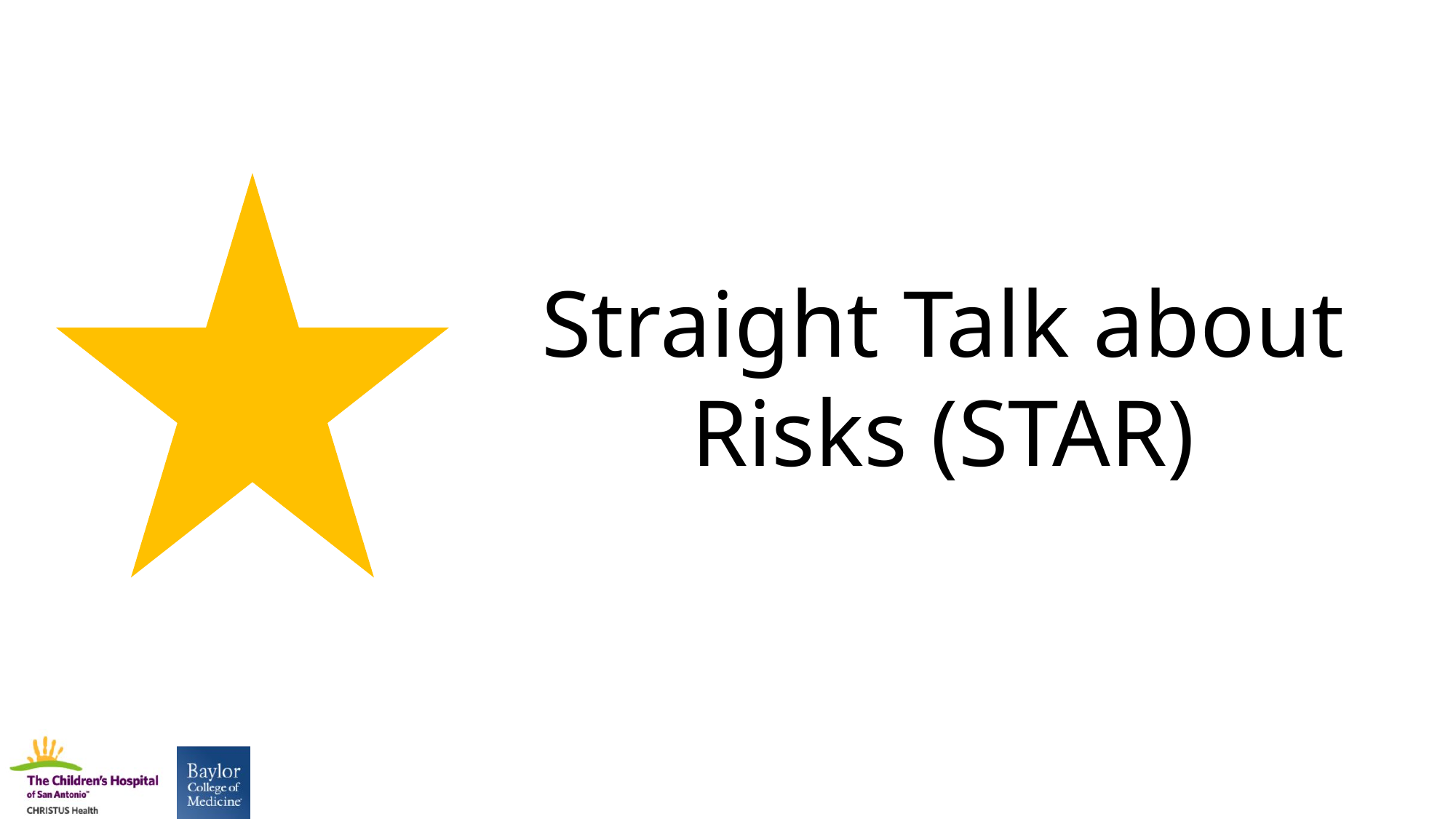

Straight Talk about Risks (STAR)

## Slide 20
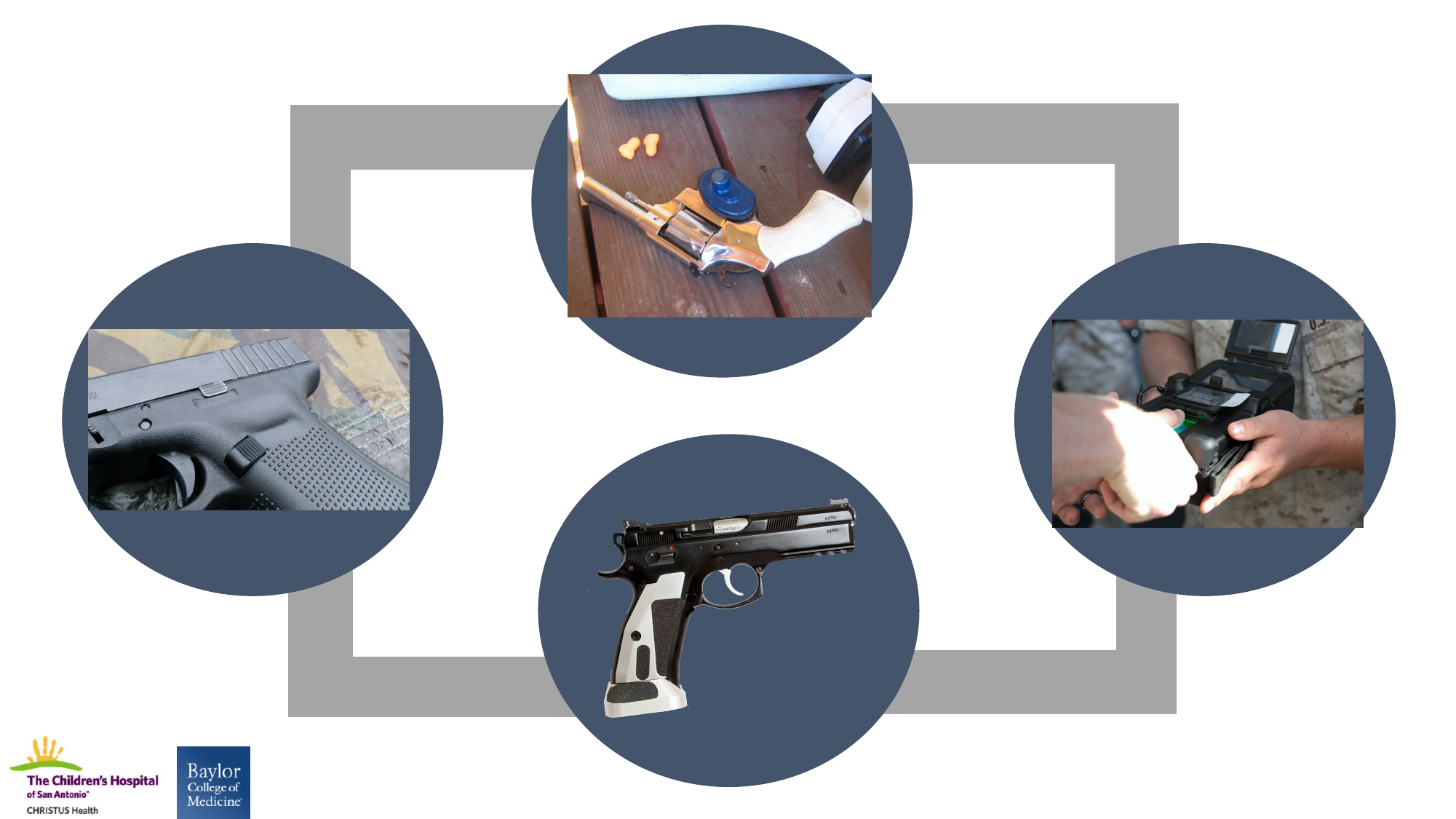

## Slide 21
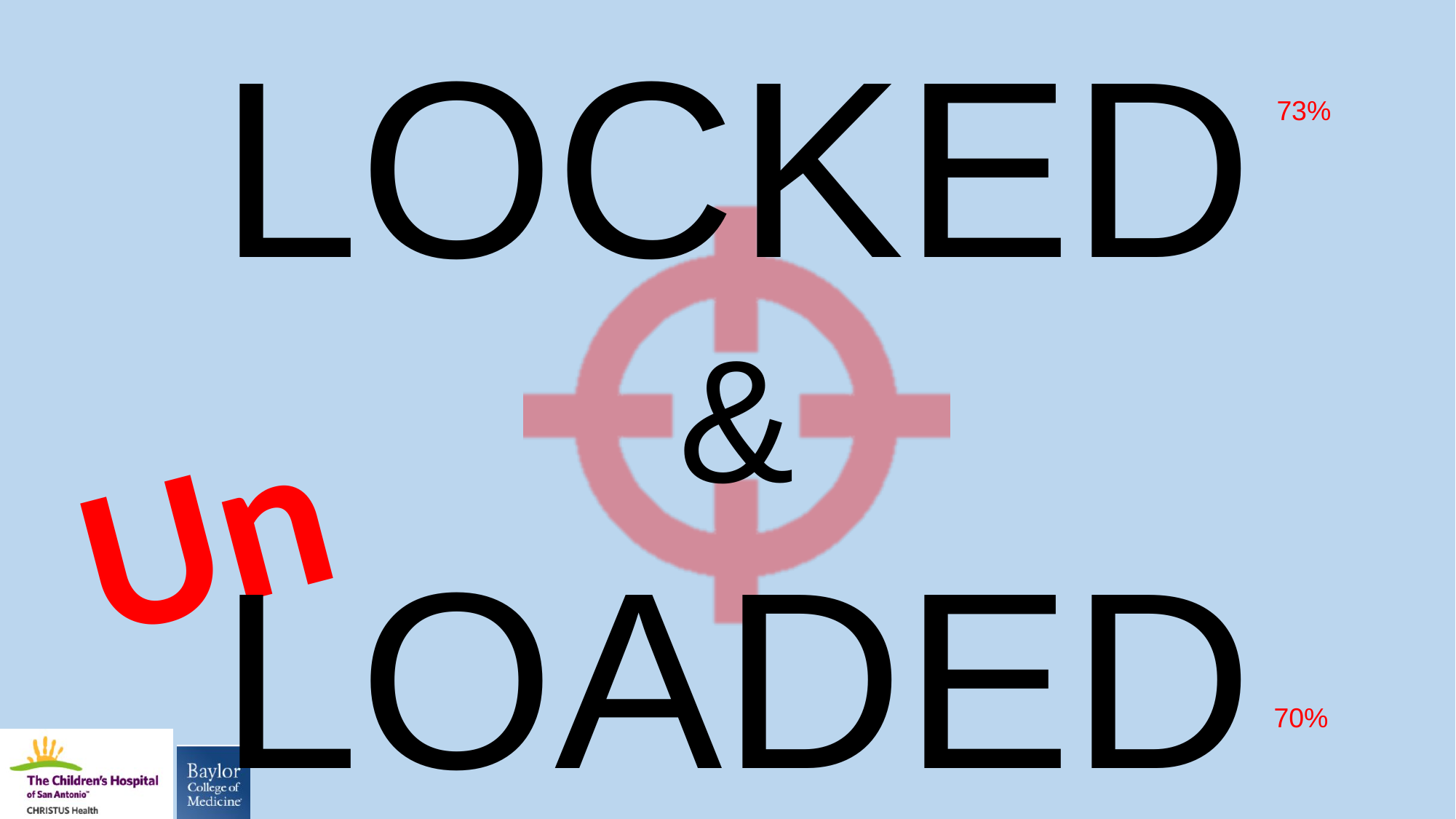

LOCKED
&
LOADED
73%
Un
70%

## Slide 22
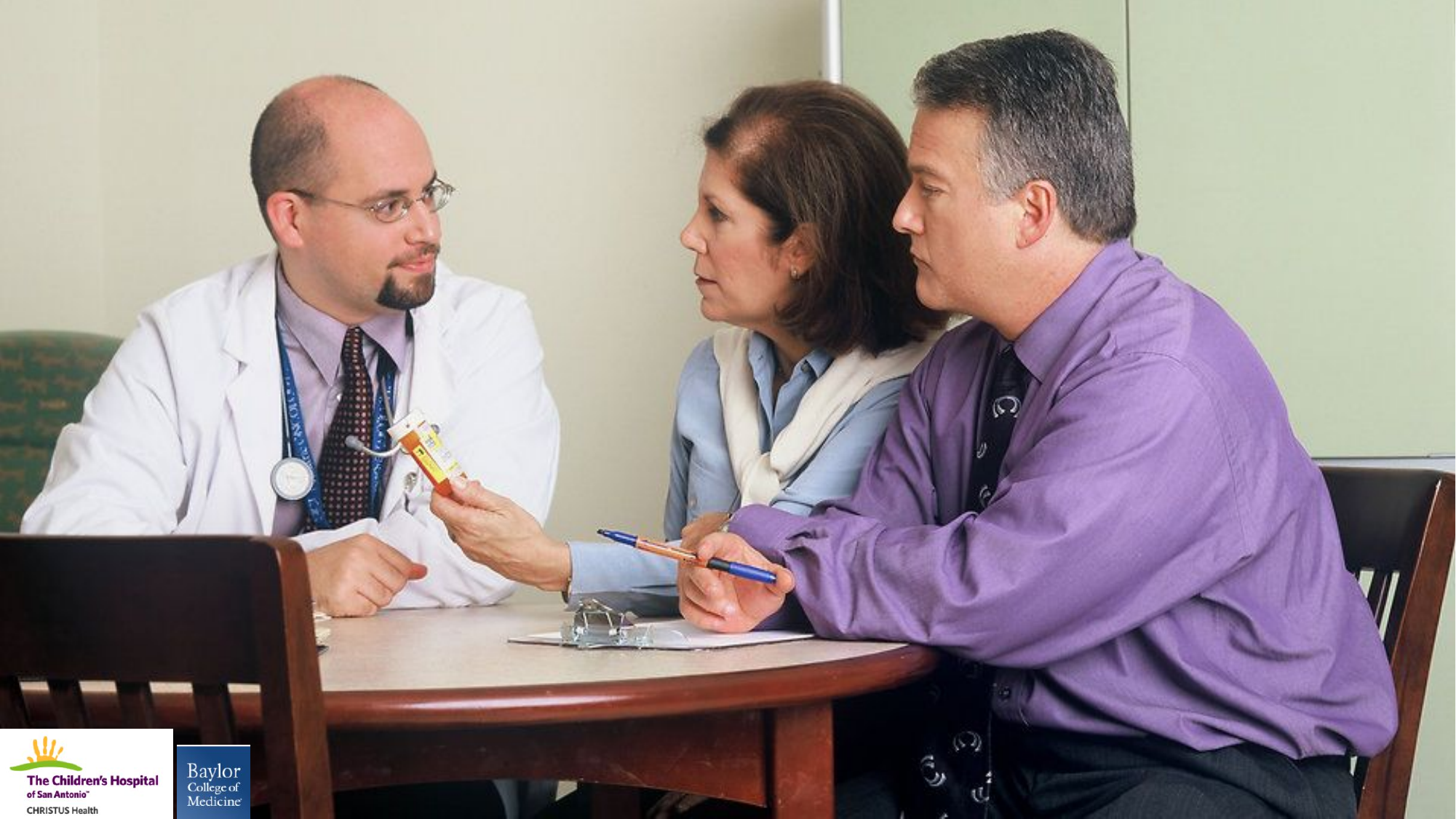

## Slide 23
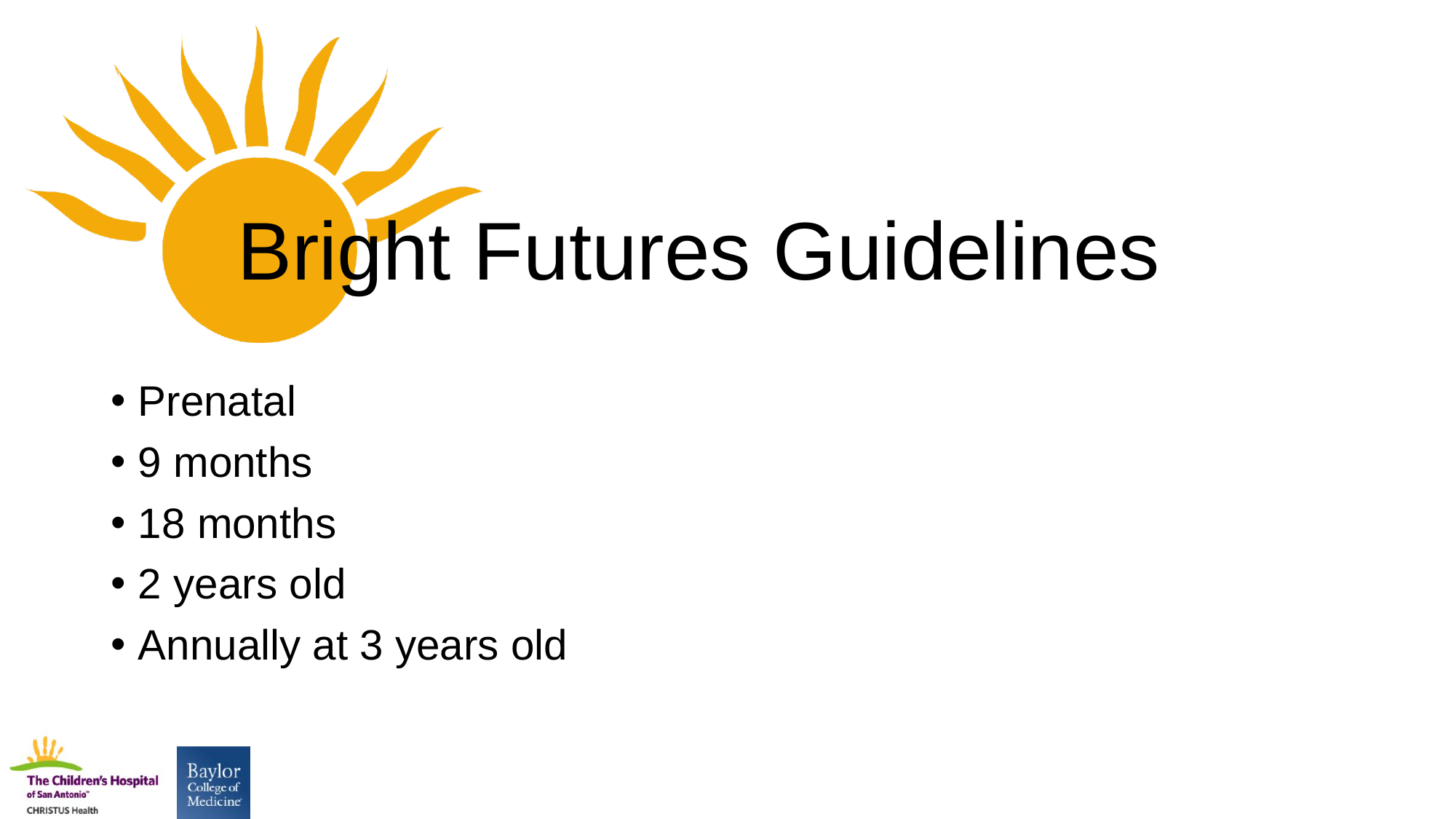

Bright Futures Guidelines
Prenatal
9 months
18 months
2 years old
Annually at 3 years old

## Slide 24
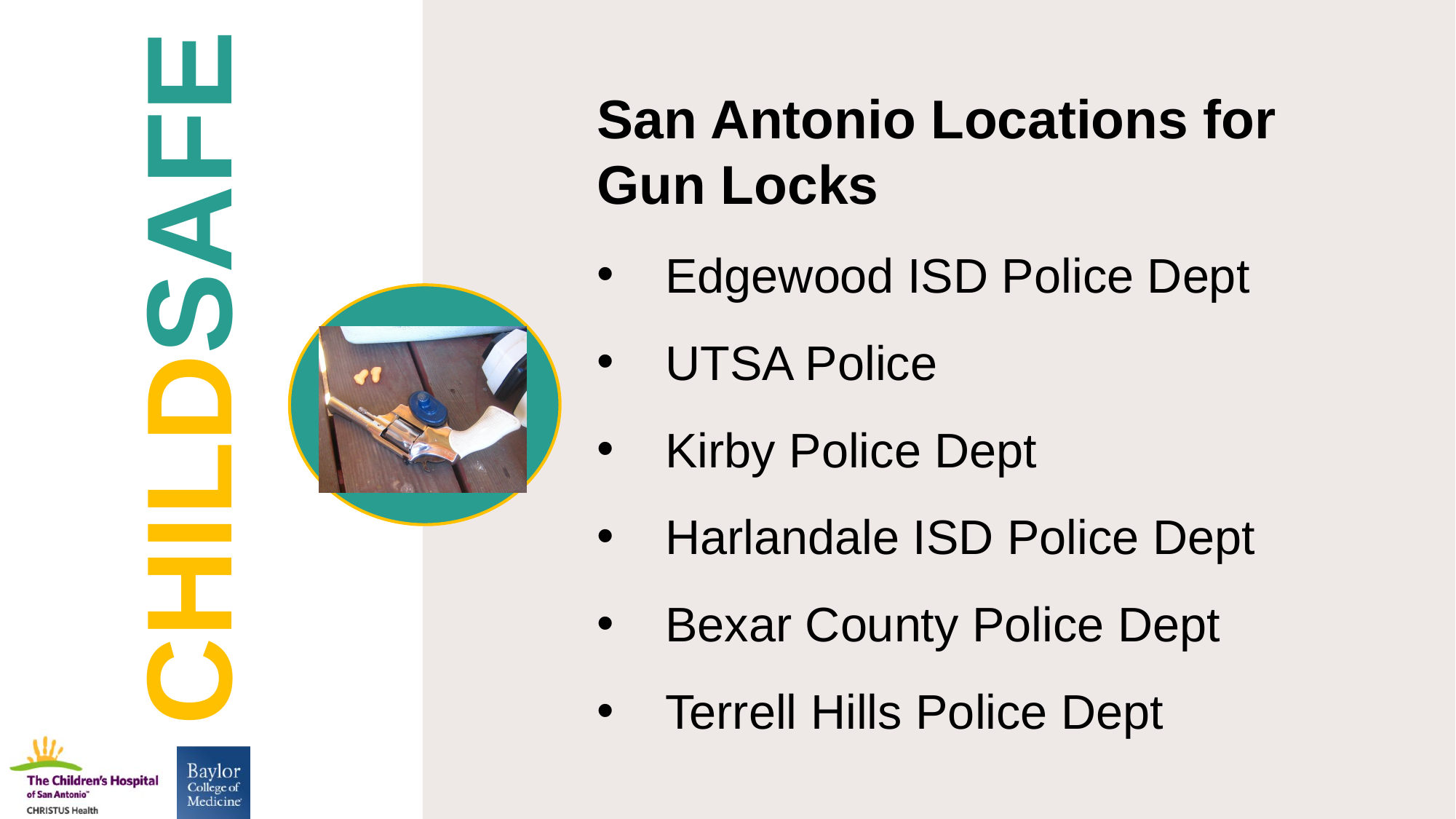

San Antonio Locations for
Gun Locks
Edgewood ISD Police Dept
UTSA Police
Kirby Police Dept
Harlandale ISD Police Dept
Bexar County Police Dept
Terrell Hills Police Dept
CHILDSAFE

## Slide 25
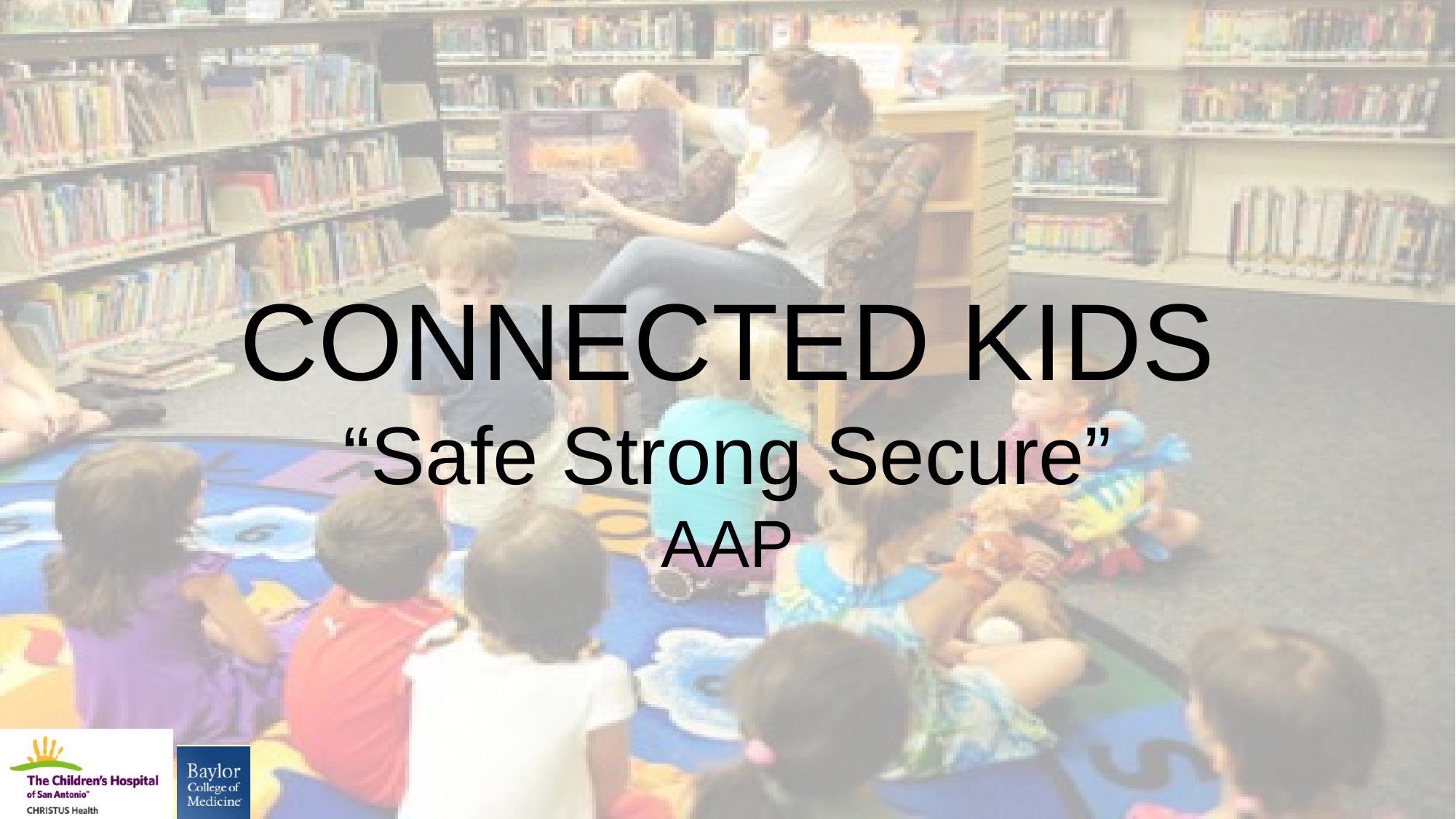

CONNECTED KIDS
“Safe Strong Secure”
AAP

## Slide 26
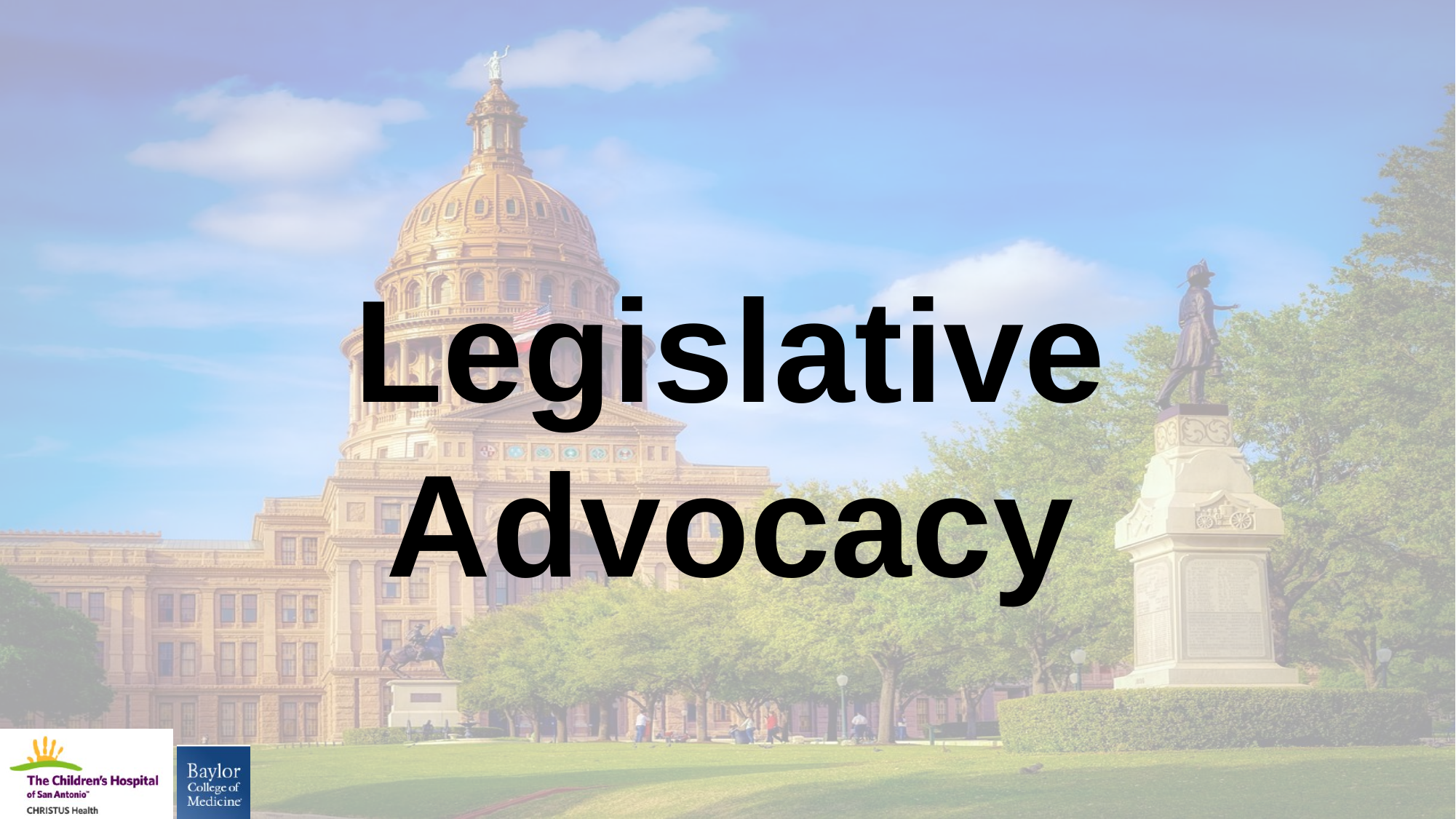

Legislative Advocacy

## Slide 27
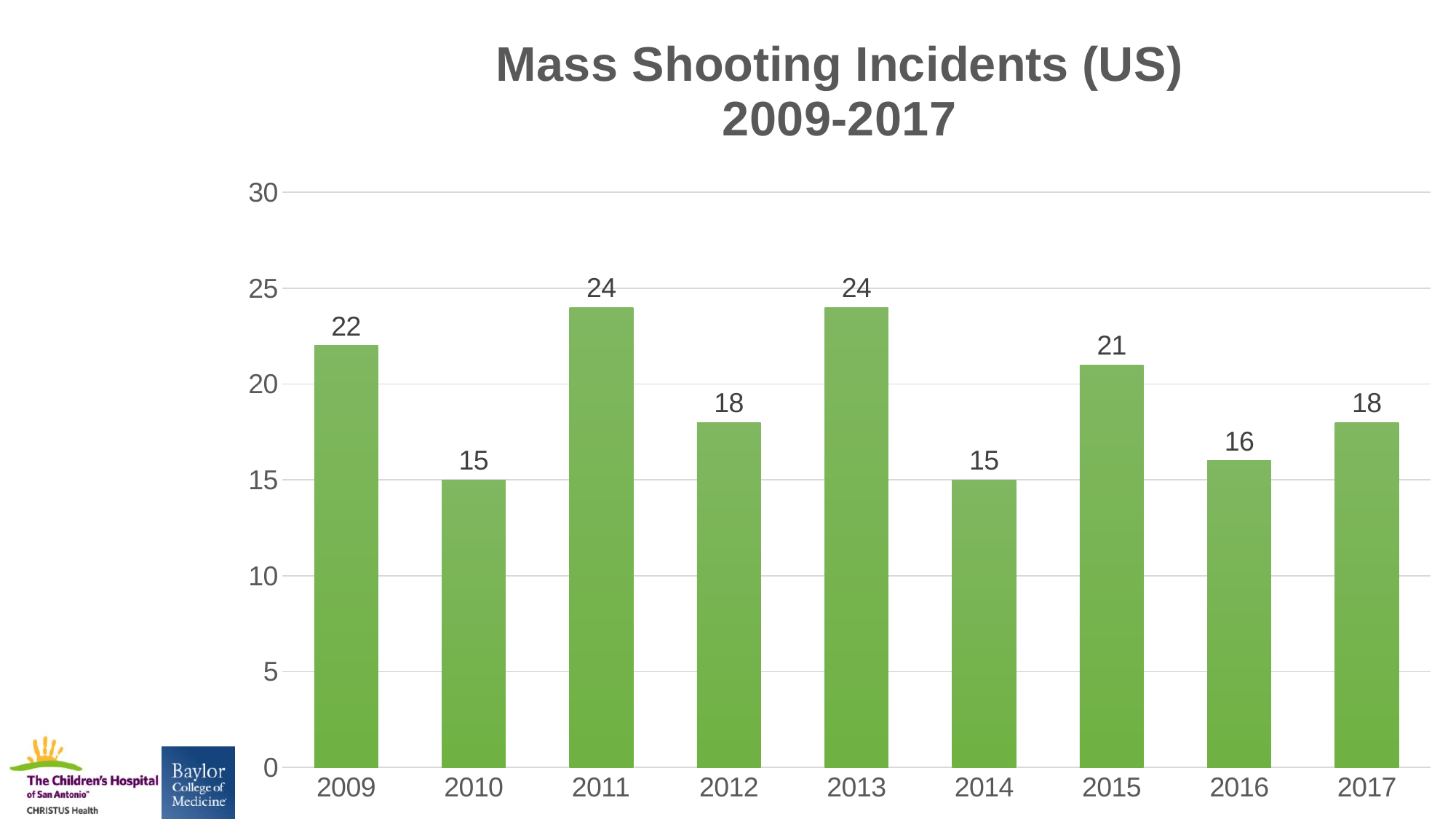

### Chart: Mass Shooting Incidents (US)
2009-2017
| Category | Mass Shooting Incidents by Year (2009-2017) |
|---|---|
| 2009 | 22.0 |
| 2010 | 15.0 |
| 2011 | 24.0 |
| 2012 | 18.0 |
| 2013 | 24.0 |
| 2014 | 15.0 |
| 2015 | 21.0 |
| 2016 | 16.0 |
| 2017 | 18.0 |

## Slide 28
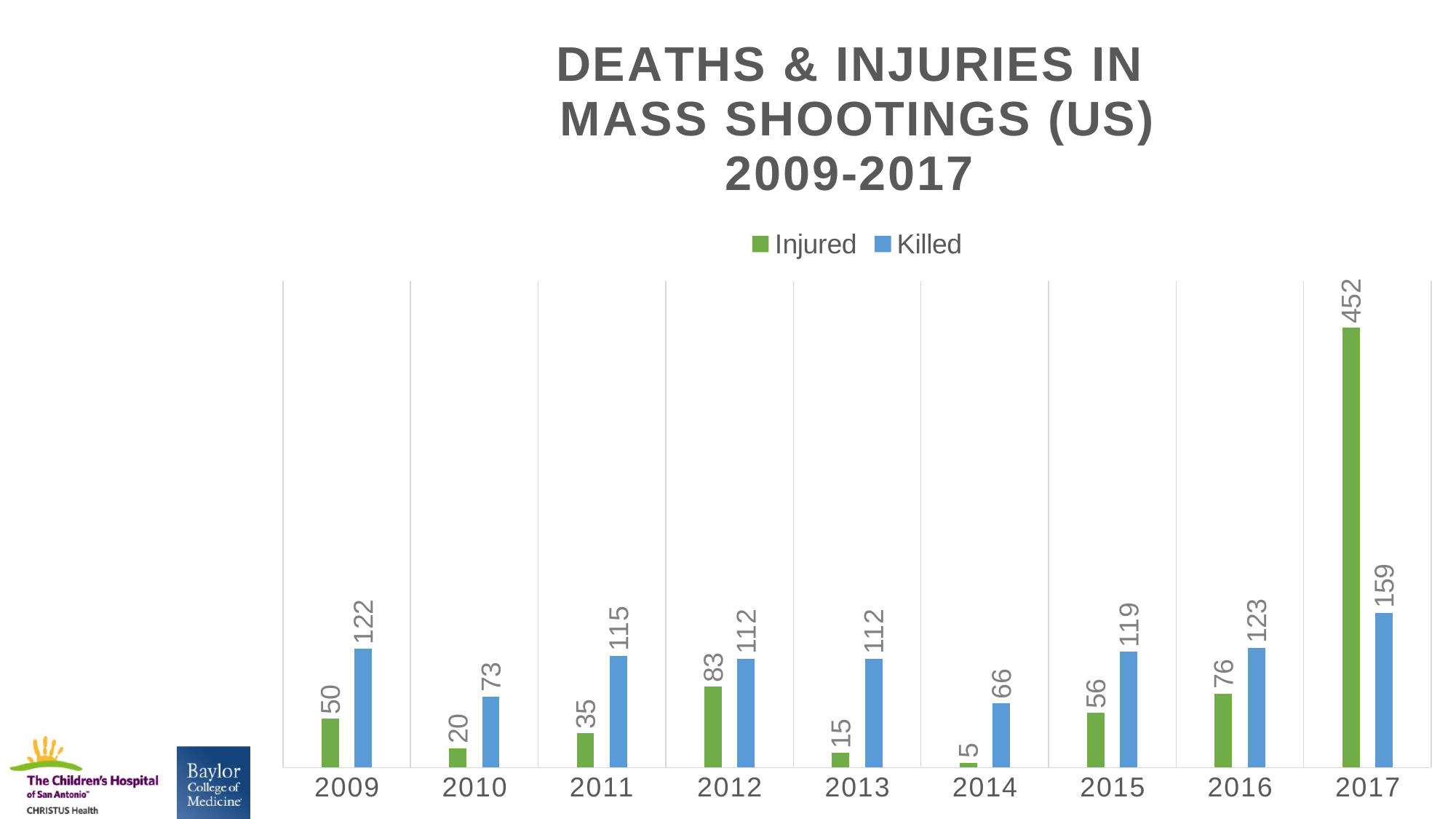

### Chart: DEATHS & INJURIES IN
MASS SHOOTINGS (US)
2009-2017
| Category | Injured | Killed |
|---|---|---|
| 2009 | 50.0 | 122.0 |
| 2010 | 20.0 | 73.0 |
| 2011 | 35.0 | 115.0 |
| 2012 | 83.0 | 112.0 |
| 2013 | 15.0 | 112.0 |
| 2014 | 5.0 | 66.0 |
| 2015 | 56.0 | 119.0 |
| 2016 | 76.0 | 123.0 |
| 2017 | 452.0 | 159.0 |

## Slide 29
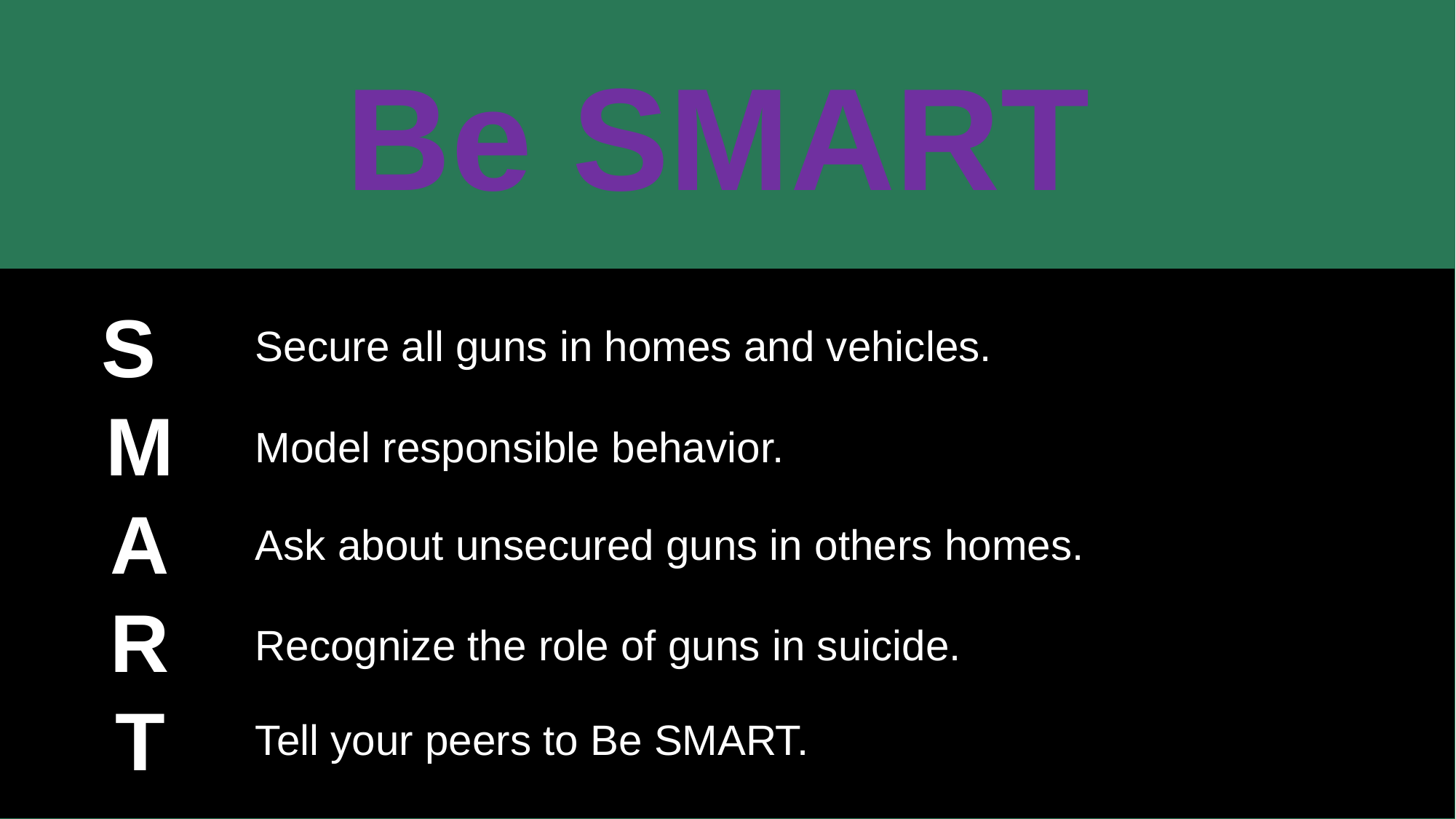

Be SMART
S
M
A
R
T
Secure all guns in homes and vehicles.
Model responsible behavior.
Ask about unsecured guns in others homes.
Recognize the role of guns in suicide.
Tell your peers to Be SMART.

## Slide 30
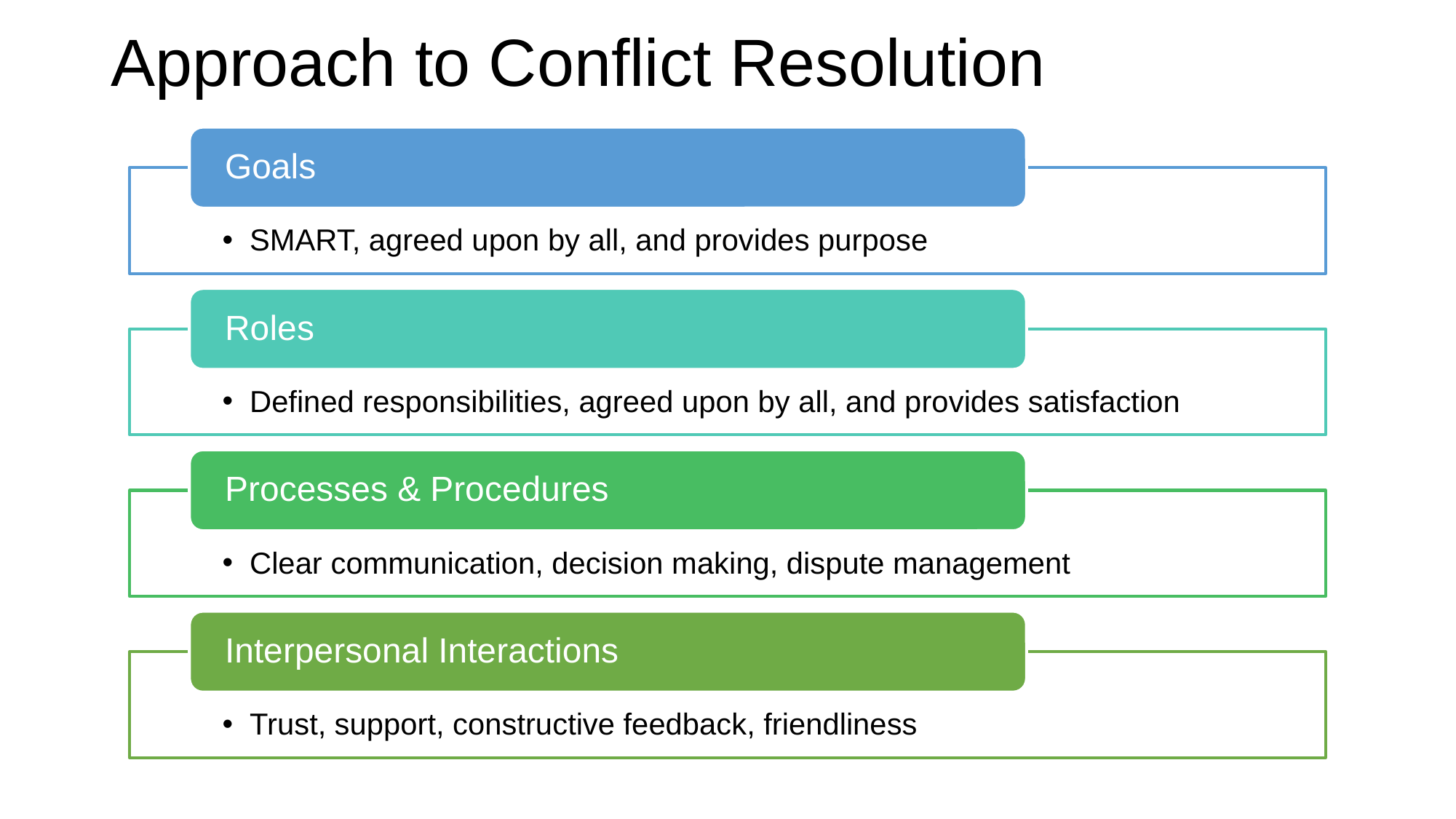

# Approach to Conflict Resolution
Goals
SMART, agreed upon by all, and provides purpose
Roles
Defined responsibilities, agreed upon by all, and provides satisfaction
Processes & Procedures
Clear communication, decision making, dispute management
Interpersonal Interactions
Trust, support, constructive feedback, friendliness

## Slide 31
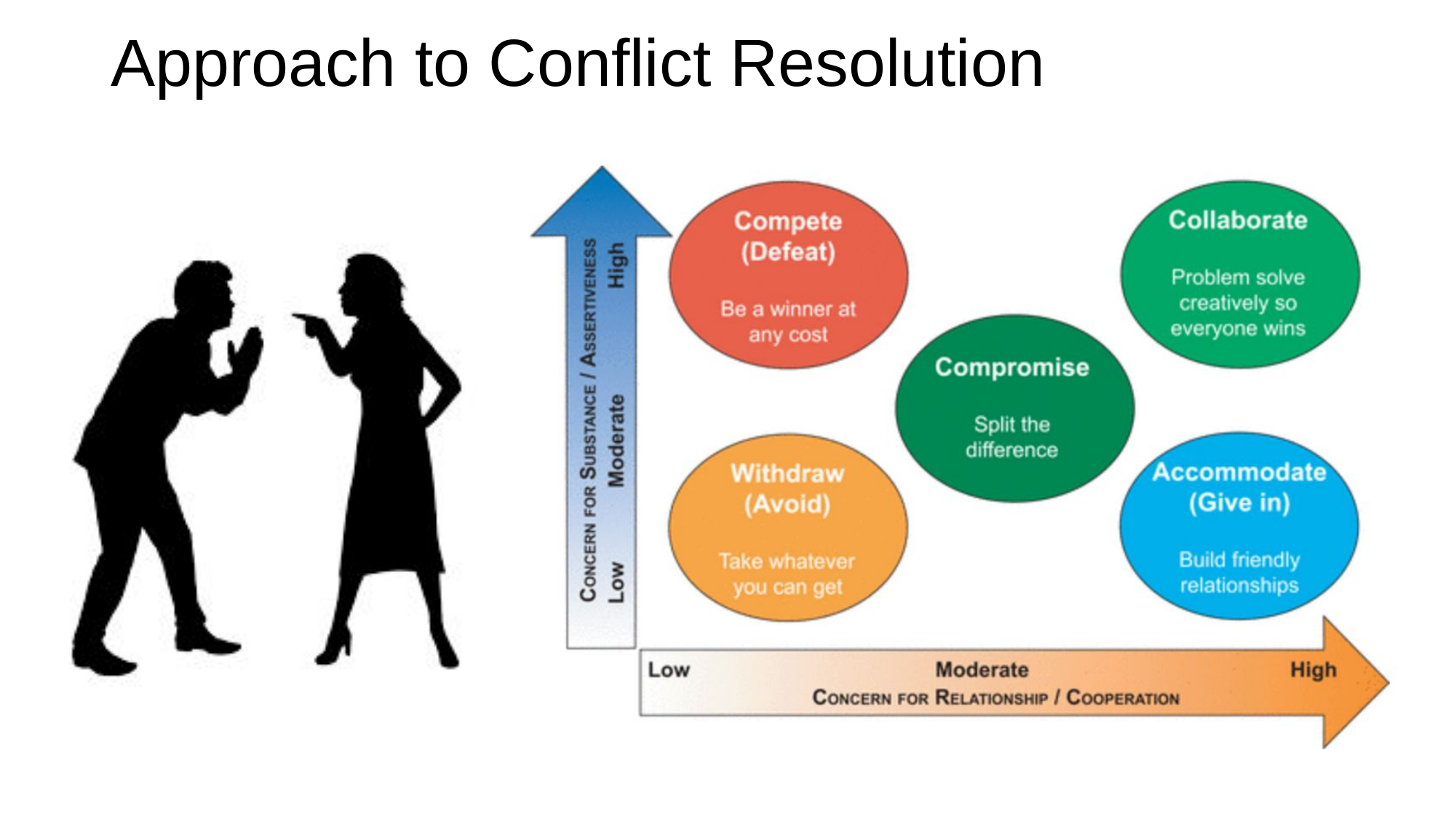

# Approach to Conflict Resolution

## Slide 32
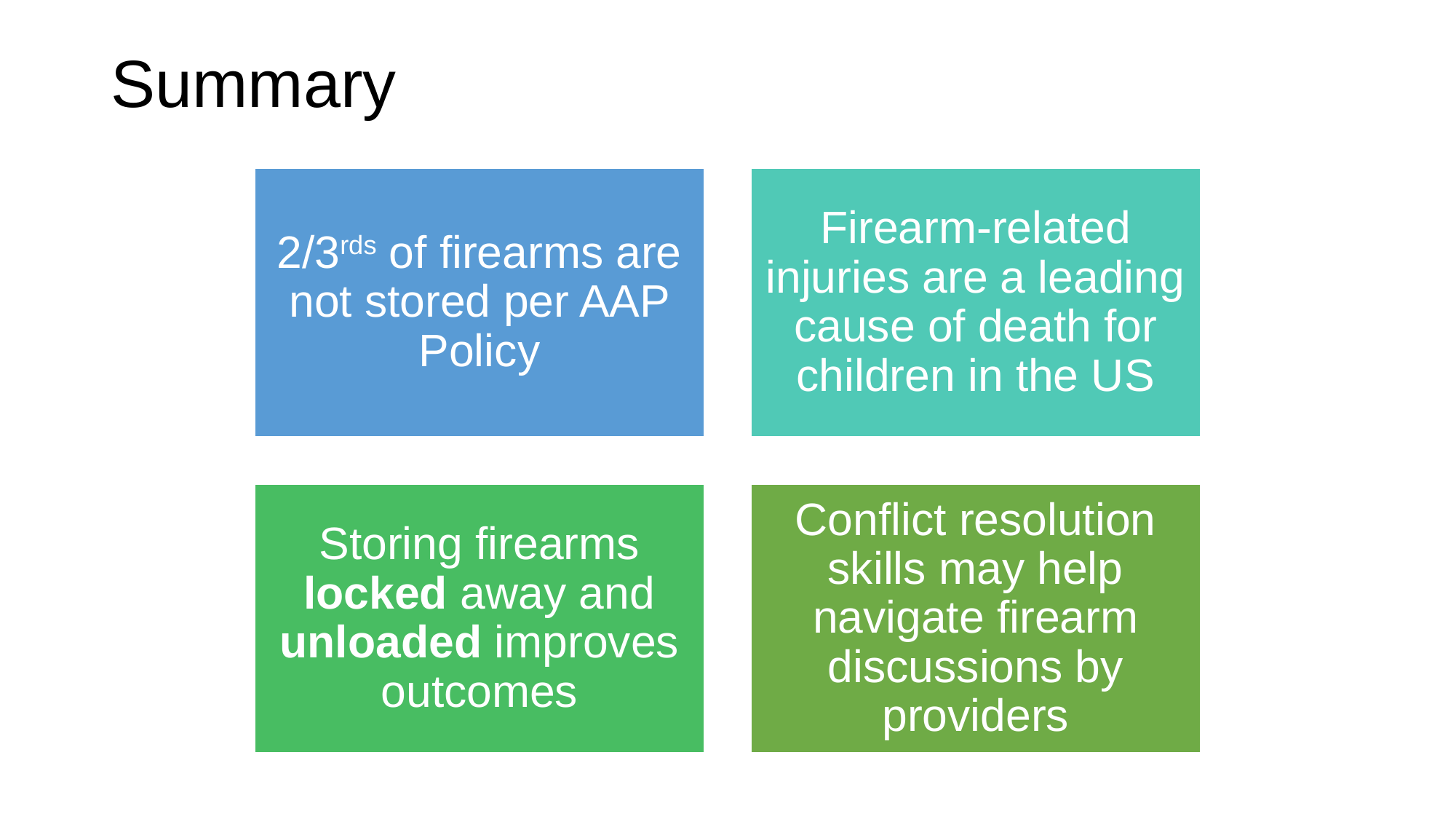

# Summary
2/3rds of firearms are not stored per AAP Policy
Firearm-related injuries are a leading cause of death for children in the US
Storing firearms locked away and unloaded improves outcomes
Conflict resolution skills may help navigate firearm discussions by providers
