# Supplementary material for: Locked and (Un)-Loaded Discussions: A Pediatric Resident Safe Firearm Storage Counseling Curriculum
Source: MedEdPORTAL. 2020 Dec 4;16:11028. doi: 10.15766/mep_2374-8265.11028 (PMC7727610; doi:10.15766/mep_2374-8265.11028)
Supplement: Supplementary file 1 — Preintervention Survey.docxDidactic Lecture.pptxFirearm & Safety-Storage Devices.mp4Sample Phone Script & Email to Law Enforcement.docxRole-Playing Scenarios.docxFacilitators Guide for Role-Playing Scenarios.docxPostintervention Survey.docxEHR Chart Audit Tool.docx [file mep_2374-8265.11028-s001.zip › D. Sample Phone Script & Email to Law Enforcement.docx]

We encourage educators to connect with either their campus or local (county or city) law enforcement to deliver the hands-on firearm and firearm storage device portion of the curriculum. We offer these phone and email scripts to invite them to do so.

**Locked and (Un)-Loaded Discussions Firearm and Safety-Storage Devices Sample Phone Script for Law Enforcement**

Hello. I am Dr. _____________. My colleagues and I are delivering a safe firearm storage counseling curriculum to our [*type of learners*]. This curriculum teaches learners how to engage in conversations with and counsel parents and caregivers about safely storing their firearms in their homes in an effort to prevent injuries. The presentation is non-partisan and not political. Would you be interested in demonstrating various firearms and storage devices during this presentation? Thank you.

**Locked and (Un)-Loaded Discussions Firearm and Safety-Storage Devices Sample Email for Law Enforcement**

Dear ______,

I am a pediatrician at __________. My colleagues and I are delivering a safe firearm storage counseling curriculum to our [*type of learners*]. This curriculum teaches learners how to engage in conversations with and counsel parents and caregivers about safely storing their firearms in their homes in an effort to prevent injuries. The presentation is non-partisan and not political. Would you be interested in demonstrating various firearms and storage devices during this presentation?

We appreciate your help with this timely and relevant issue.

Sincerely,

[Your Name and Contact Information]
