# Supplementary material for: Locked and (Un)-Loaded Discussions: A Pediatric Resident Safe Firearm Storage Counseling Curriculum
Source: MedEdPORTAL. 2020 Dec 4;16:11028. doi: 10.15766/mep_2374-8265.11028 (PMC7727610; doi:10.15766/mep_2374-8265.11028)
Supplement: Supplementary file 1 — Preintervention Survey.docxDidactic Lecture.pptxFirearm & Safety-Storage Devices.mp4Sample Phone Script & Email to Law Enforcement.docxRole-Playing Scenarios.docxFacilitators Guide for Role-Playing Scenarios.docxPostintervention Survey.docxEHR Chart Audit Tool.docx [file mep_2374-8265.11028-s001.zip › E. Role-Playing Scenarios.docx]

**Safe Firearm Storage Counseling Scenario 1**

Role: Parent

You are the parent of Pedro, who is seeing his pediatrician for a 4-year well child care visit. Pedro does not have any medical issues and is doing well overall. He lives with you, his father, 7 year-old sister, and 14 year-old brother. During the safety portion of the interview, you are asked if you have any guns in your home and you quickly respond, “no.” The pediatrician then asks you a few other questions.

Potential Topics of Concern:

- You are strongly considering purchasing a gun in the next few weeks.
- Your 14 year-old son was just diagnosed with depression and has started taking an antidepressant less than 1 week ago.

**Safe Firearm Storage Counseling Scenario 1**

Role: Pediatrician

You are a pediatrician seeing Pedro, who is here for his 4-year well child care visit. Pedro does not have any medical issues and is doing well overall. He lives with his mother, father, 7 year-old sister, and 14 year-old brother. During the safety portion of the interview, you ask the family if they have any guns in the home and they quickly respond, “no.” Is this the end of this topic or would you like to ask any other questions?

Potential Next Questions:

- Would you like to know how to safely store a gun in the case you obtain one?
- Would you like to know what the AAP recommends regarding guns and gun safety?
- Would you like any advice on how to educate Pedro on guns and gun safety?
- Does Pedro visit the home of anyone who has a gun? Do you know if they are properly stored?
- Does anyone who is frequently around Pedro have a license to carry a gun?
- Do his siblings (especially the 14 year-old) have any mental health issues and access to guns?

**Safe Firearm Storage Counseling Scenario 2**

Role: Parent

Carina is your 6 year-old daughter who is seeing her pediatrician today for a well child care visit. The clinic nurse has already obtained the review of systems and assessed for concerns. You (Carina’s father), Carina’s mom, and Carina have no concerns to address today. During the well child review of systems, you endorse having a firearm in your home. You own a firearm to protect your family. You do not believe your child would ever handle the firearm. You are initially resistant to safely storing your firearm as you own it to protect your family. Additionally, Carina visits her cousins frequently throughout the week and her uncle and aunt own several guns.

**Safe Firearm Storage Counseling Scenario 2**

Role: Pediatrician

Carina is a 6 year-old female who presents to the clinic with her parents for a well child care visit. The clinic nurse has already obtained the review of systems and assessed for concerns. Carina and her parents have no concerns to address today. During the well child review of systems, the father endorses having a firearm in the home. You inquire further about their storage technique.

Potential Next Steps:

- If the father is resistant to your raising the topic, how might you explain your probing? What would you say to prevent an argument?
- How might you convince the family to store the firearm as recommended by the AAP?
- How would you counsel these parents if you learn that Carina visits her cousins frequently throughout the week and her uncle and aunt own several guns?

**Safe Firearm Storage Counseling Scenario 3**

Role: Parent

Michael, your 15 year-old son who is interested in joining the military, is seeing his pediatrician today for a well child care visit. You (Michael’s father) recently retired from the military and accompany Michael for the visit. Michael is healthy, doing very well in school, and you have just returned to the room after the pediatrician conducted an interview alone with Michael. When you return, the pediatrician asks you if you have a gun in your home. You become defensive and tell the pediatrician that she doesn’t have any right to ask this question.

**Safe Firearm Storage Counseling Scenario 3**

Role: Pediatrician

You are seeing Michael, a 15 year-old boy who is interested in joining the military, for a well child care visit. Michael’s father is recently retired from the military and accompanies Michael for the visit. Michael is healthy, doing very well in school, and has a reassuring HEADSS exam that you performed prior to allowing the father to return to the room. When he does return, you ask the father if the family has a gun in the home.

Potential Next Questions

- What may have gone wrong in this scenario?
- What can you do to avert the impending argument that is likely to ensue?
- Is it appropriate for Michael to know there is a gun in his home?
