# Supplementary material for: Locked and (Un)-Loaded Discussions: A Pediatric Resident Safe Firearm Storage Counseling Curriculum
Source: MedEdPORTAL. 2020 Dec 4;16:11028. doi: 10.15766/mep_2374-8265.11028 (PMC7727610; doi:10.15766/mep_2374-8265.11028)
Supplement: Supplementary file 1 — Preintervention Survey.docxDidactic Lecture.pptxFirearm & Safety-Storage Devices.mp4Sample Phone Script & Email to Law Enforcement.docxRole-Playing Scenarios.docxFacilitators Guide for Role-Playing Scenarios.docxPostintervention Survey.docxEHR Chart Audit Tool.docx [file mep_2374-8265.11028-s001.zip › F. Facilitators Guide for Role-Playing Scenarios.docx]

**Facilitator's Guide for Safe Firearm Storage Counseling Scenarios**

We designed this Facilitator’s Guide for use during the debriefing of the Role-Playing portion of the Safe Firearm Storage Counseling curriculum. The Facilitator’s Guide includes learning objectives for each scenario and fast facts to help the learner engage in evidence-based and meaningful conversations with families.

Safe Firearm Storage Counseling Scenario 1

*Learning Objectives*

Following completion of role play with Scenario 1, learners will be able to:

- Ask about the presence of firearms in families’ homes.
- Craft gently probing questions to secure the opportunity to counsel families regardless of the presence of firearms in the home, in the event that a change should occur between health supervision visits.
- Recognize the risks of a child finding a firearm in others’ homes.
- Recall the increased risk of injury/suicide among children and teens with DSM-V diagnoses who live in homes with firearms and apply this knowledge in a clinical setting.

*Fast Facts*

- When surveyed, families assert that physician’s asking and counseling on firearm safety is well within their scope of practice.
- Of all the homes in the US with children living in them, about 33% will have a firearm. 66% of those firearms will be poorly stored, meaning they are not locked away unloaded with the ammunition locked away separately.
- Simply having a firearm in the home has been shown to increase the risk of suicide for adolescents, even if they do not carry a DSM-V diagnosis for a mood disorder! However, with a DSM-V diagnosis, the risk is increased substantially. This risk can be significantly decreased by removal of the firearm from the home or storage according to the AAP policy statement.

Safe Firearm Storage Counseling Scenario 2

*Learning Objectives*

Following completion of role play with Scenario 2, learners will be able to:

- Clarify the cause and risk of injury during a home invasion in a home with a firearm.
- Contrast the risk of injury to a child in a home with an unsecured firearm as compared to during a home invasion.
- Explain to parents/caregivers the curious nature of children to handle firearms when found.
- Navigate and de-escalate a difficult conversation in a non-judgmental fashion to affect a positive outcome, highlighting the mutual goal of child safety.

*Fast Facts*

- It is a common misconception that use of a firearm during a home invasion will have a protective effect. A study reviewing individuals who had experienced a home invasion in the last year actually showed that those individuals who used a firearm were 4 times more likely to be shot during the home invasion!
- Several firearm injury prevention programs utilize behavioral training in children to prevent interacting with a firearm if accidentally found. To date, this intervention, which suggests teaching the child to stop, run away, and tell an adult, has not been shown to be effective in decreasing fire-arm related injuries. In one study, a child was actually more likely to play with a firearm when accidentally found after having undergone behavioral training.
- Conflict resolution is a skill to be learned that involves balancing your willingness to cooperate with others alongside your desire for assertiveness. An equal balance is demonstrated through the collaborative and possibly compromising conflict resolution styles. Remaining calm and emphasizing a mutual goal of child safety can help the conversation remain in a collaborative and/or compromising state.

Safe Firearm Storage Counseling Scenario 3

*Learning Objectives*

Following completion of role play with Scenario 3, learners will be able to:

- Utilize conflict resolution strategies to engage in a factual, unemotional conversation about unsecured firearms in the home.
- Recognize when further discussion may prove futile and only damage a physician-patient/family relationship.
- Discuss state laws and parent/caregiver liability should a child or teen discharge a firearm owned by the parent/caregiver.

*Fast Facts*

- Parents and caregivers may or may not choose to inform children and teens of the presence of firearms in the home; however, organizations that promote child safety and injury prevention (eg. American Academy of Pediatrics, Project Child Safe, National Shooting Sports Foundation) encourage parents/caregivers to talk to their children about what to do if they find a firearm (i.e. assume it is real, assume it is loaded, do not handle the gun, leave and find an adult).
- Individual state Child Access Prevention laws dictate parent/caregiver liability in the event that a child discharges a firearm owned by the parent/caregiver. For state-by-state policy summaries, see <https://lawcenter.giffords.org/gun-laws/state-law/50-state-summaries/child-access-prevention-state-by-state/>.
