# Supplementary material for: Locked and (Un)-Loaded Discussions: A Pediatric Resident Safe Firearm Storage Counseling Curriculum
Source: MedEdPORTAL. 2020 Dec 4;16:11028. doi: 10.15766/mep_2374-8265.11028 (PMC7727610; doi:10.15766/mep_2374-8265.11028)
Supplement: Supplementary file 1 — Preintervention Survey.docxDidactic Lecture.pptxFirearm & Safety-Storage Devices.mp4Sample Phone Script & Email to Law Enforcement.docxRole-Playing Scenarios.docxFacilitators Guide for Role-Playing Scenarios.docxPostintervention Survey.docxEHR Chart Audit Tool.docx [file mep_2374-8265.11028-s001.zip › H. EHR Chart Audit Tool.docx]

**EHR Chart Audit Tool**

We collaborated with representatives from our Electronic Health Record team to insert a “firearm safety” field within the Review of Systems section of well child care encounters. When clicked, the field is highlighted, signifying that the provider initiated a discussion about firearm safety. When double clicked, the field is highlighted and an adjacent text box appears, allowing the provider to expand on details of the discussion.

We used the following simple chart audit tool to collect provider-specific safe firearm storage counseling data during well child care encounters for children > 3 years.*

Date of Visit: __/__/____

Name of Provider: _____________

Was the patient seen for a well child care visit?: ___Yes ___No

Is the patient 3 years or older?: ___Yes ___No

Was firearm safety discussed during the visit?: ___Yes ___No

*Although American Academy of Pediatrics Bright Futures Guidelines recommend counseling families regarding safe firearm storage earlier than age 3, following a rich and engaging discourse among primary care pediatricians across our institution, a decision was made to add the “firearm safety” field to the Review of Systems section of well child care encounters for children > 3 years.
